# Construction and Multiple Feature Classification Based on a High-Order Functional Hypernetwork on fMRI Data

**DOI:** 10.3389/fnins.2022.848363

**Published:** 2022-04-13

**Authors:** Yao Li, Qifan Li, Tao Li, Zijing Zhou, Yong Xu, Yanli Yang, Junjie Chen, Hao Guo

**Affiliations:** ^1^College of Information and Computer, Taiyuan University of Technology, Taiyuan, China; ^2^College of Software, Taiyuan University of Technology, Taiyuan, China; ^3^Department of Psychiatry, First Hospital of Shanxi Medical University, Taiyuan, China

**Keywords:** high-order functional network, multi-feature extraction, multi-kernel learning, fMRI, classification, depression, hypernetwork

## Abstract

Resting-state functional connectivity hypernetworks, in which multiple nodes can be connected, are an effective technique for diagnosing brain disease and performing classification research. Conventional functional hypernetworks can characterize the complex interactions within the human brain in a static form. However, an increasing body of evidence demonstrates that even in a resting state, neural activity in the brain still exhibits transient and subtle dynamics. These dynamic changes are essential for understanding the basic characteristics underlying brain organization and may correlate significantly with the pathological mechanisms of brain diseases. Therefore, considering the dynamic changes of functional connections in the resting state, we proposed methodology to construct resting state high-order functional hyper-networks (rs-HOFHNs) for patients with depression and normal subjects. Meanwhile, we also introduce a novel property (the shortest path) to extract local features with traditional local properties (cluster coefficients). A subgraph feature-based method was introduced to characterize information relating to global topology. Two features, local features and subgraph features that showed significant differences after feature selection were subjected to multi-kernel learning for feature fusion and classification. Compared with conventional hyper network models, the high-order hyper network obtained the best classification performance, 92.18%, which indicated that better classification performance can be achieved if we needed to consider multivariate interactions and the time-varying characteristics of neural interaction simultaneously when constructing a network.

## Introduction

Over recent years, the use of neuroimaging technology to investigate the interaction of brain regions has gained increasing levels of attention. For example, the Blood Oxygen Level-Dependent (BOLD) signal is now routinely used as a neurophysiological indicator for resting-state functional magnetic resonance imaging (rs-fMRI) to detect endogenous or spontaneous activity in the brain neurons (Burrows et al., 2021). Based on BOLD signals, a functional connectivity network can be constructed and then used to investigate the pathological mechanisms underlying brain diseases. This methodology has been successfully applied in the diagnosis of schizophrenia (Steardo et al., 2020), attention deficit syndrome (Riaz et al., 2020), depression (Porta-Casteràs et al., 2021), Alzheimer’s disease (Shao et al., 2020).

Most of the existing studies involving functional connectivity networks adopted pairwise correlation-based methods to characterize the interaction between two brain regions (Bullmore and Sporns, 2009; Jie et al., 2014). However, previous studies found that brain regions may directly interact with multiple other regions of the brain in neurological processes (Shuai et al., 2010). Moreover, recent studies have shown that there are obvious higher-order interactions in the real activity of neurons, including neuron isotope tracing, local field potentials, and cortical activity (Glickfeld and Olsen, 2017; Montangie and Montani, 2017; Baravalle and Montani, 2020). Therefore, based on neurological findings, pairwise correlation analysis may be inaccurate with regards to revealing the active cognitive activities of the brain. This type of interaction among multiple brain regions, that is, high-level information, may be critical for studying the underlying pathological basis.

Considering these problems, researchers have suggested that hyper-networks may be able to express interactive information from multiple brain areas (Jie et al., 2016). Hyper-networks are based on hypergraph theory, in which an edge can connect an arbitrary number of nodes; in other words, a hyper-edge can represent a specific relationship between multiple objects (Battiston et al., 2020). In the field of neuroimaging, the nodes in a functional hyper-network represent specific brain regions while the hyper-edges represent informational interaction among brain regions. In the past few years, hypergraphs have been widely employed in a range of medical imaging fields, including image segmentation (Dong et al., 2015) and classification (Gao et al., 2015; Liu et al., 2016). In a previous study, Jie et al. (2016) used the Least absolute shrinkage and selection operator (LASSO) method to create a hyper-network model and applied this to the diagnosis of brain diseases. In another study, Yang et al. (Li et al., 2017) adopted the star expansion method to construct structural hyper-networks and functional hyper-networks, respectively, to then perform classification analysis. Taking into account the group effecting problem within brain networks, Guo et al. (2018a) proposed the elastic net and group LASSO method to improve the establishment of hyper-network models to facilitate brain disease classification research. Considering the information featured in different time resolution fMRI, Yang et al. (Li Y. et al., 2019) proposed a functionally weighted LASSO method to build a multi-modal functional hyper-network; results showed that this model achieved better classification performance. In another study, Li et al. (2020) further considered the group structure problem associated with brain regions, and proposed the sparse group LASSO method to construct a brain functional hyper-network which was then used to study the classification of brain diseases. Subsequently, Wang et al. (2018) created a hyper-network to characterize brain connectivity information based on the LASSO method and combined this with network voxel information to investigate the relationship between brain network features and genetic variation. In another study, Gu et al. (2017) reported a hypergraph representation method using BOLD rs-fMRI data which divided the hyperedge into three different categories (bridges, stars, and clusters) to represent the binary, focus, and spatial distribution of architecture, respectively. Xiao et al. (2020) constructed a weighted hyper-network based on the sparse representation method and the hypergraph learning method, and used this to classify personal learning ability.

These functional brain hyper-network models usually capture interactions between multiple brain regions in a static form. In other words, in the resting state brain function network, the functional connections remain unchanged over time. However, increasing evidence suggests that even in the resting state, the neural activity in the brain still exhibits transient and subtle dynamics (Kudela et al., 2017; Zhao et al., 2020). Moreover, these dynamic changes are essential for understanding the basic characteristics relating to brain organization and may be significantly correlated with the pathological mechanisms underlying brain diseases; consequently, these changes may provide useful information for disease classification (Kudela et al., 2017; Zhao et al., 2020). Therefore, considering the dynamic changes of functional connections in the resting state, we proposed the construction of a resting state high-order functional hyper-network (rs-HOFHN) to simultaneously reflect the temporal dynamics of working mechanism with the human brain and the multiple interaction of space.

The extension of methods to study time-varying connectivity in the brain has emerged along multiple lines, including the detection of important transition points, for example, change-point analysis (Cribben et al., 2012), time-frequency approaches (Chang and Glover, 2010), and windowing approaches (Calhoun et al., 2014; Vidaurre et al., 2017; Yu et al., 2018). Of these, the sliding time window is a popular approach for validating dynamic functional connectivity in fMRI data across a short period of time (Calhoun et al., 2014; Vidaurre et al., 2017; Yu et al., 2018). Thus, we used the sliding time window method to reflect time-variable connectivity in the brain.

In previous studies of brain functional hypernetworks, researchers usually used a single type of quantifiable property, for example, cluster coefficients (Jie et al., 2016; Li et al., 2017; Guo et al., 2018a; Wang et al., 2018; Li Y. et al., 2019). Although only a single property is used to obtain better classification performance, this method ignores the role of other properties in the hypernetwork. This makes the expression of the hypernetwork topology information one-sided and flat; in turn, this affects the effectiveness of the classification model (Xiao, 2013). Thus, we introduced a new property, shortest path length (Zhang and Liu, 2010), into our neuroimaging research and combined this with the traditional clustering coefficient to evaluate local topology information in the high-order functional hypernetwork from multiple angles. In addition, studies have shown that if only local feature properties were used to characterize local topology information in the hypernetwork, some important topology information would still be lost to a certain extent, such as global topology information in the brain network (Wang et al., 2015). Considering this problem, we introduced hyperedges as subgraph features to characterize global topological information of the high-order brain function hypernetwork.

Specifically, we used the sliding time window method (Yu et al., 2018) to obtain a relevant time series. Based on the relevant time series, the sparse group LASSO method (Li et al., 2020) was used to construct a high-order brain hyper-network. We then introduced local topological properties and subgraph features to reflect the complete topology of the high-order brain functional hyper-network, thus providing more accurate and relevant imaging markers. Specifically, two different types of clustering coefficients, and the shortest path, was then introduced to extract node information to represent connectivity information of the brain function hyper network and reflect the separation and integration characteristics of local brain activities. Next, local difference features were selected using non-parametric tests. The hyperedges were used as subgraph features to represent the global topology information in the brain network (Wang et al., 2015); then, we used the frequently scoring feature selection (FSFS) method to select discriminant subgraphs. Finally, multi-kernel learning was introduced to fuse the two types of features and a construct classification method.

The main aims of this study were to (1) construct a high-order functional hyper-network by applying the sliding time window and sparse group LASSO method; (2) extract local features by using multiple types of local properties that characterize the network local topology of the high-order hyper-network and extract key features by non-parametric analysis; (3) extract subgraph features by using hyperedges that characterize the global topology information provided by the high-order hyper-network and select discriminative features using the FSFS algorithm, and (4) use multi-kernel learning to fuse the two types of features and perform classification. The classification results showed that compared with the conventional hyper-network model, the high-order hyper network achieved better classification performance. In addition, we analyzed the network topology of the high-order functional hypernetwork and the biological significance of the different brain areas obtained by the high-order hypernetwork. Moreover, we analyzed the influence of key model and classifier parameters on classification performance.

## Materials and Methods

### Method Framework

**FIGURE 1:**
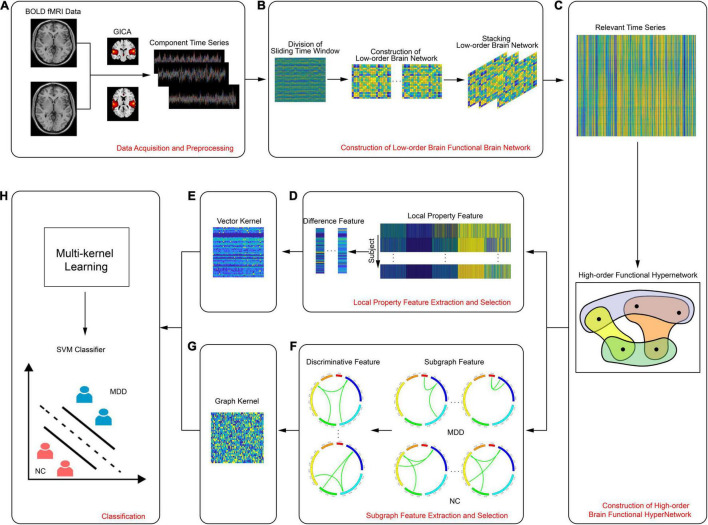
Flowchart showing the experimental process, including **(A)** data acquisition and preprocessing, **(B)** construction of low-order brain functional brain network, **(C)** construction of high-order brain functional hypernetwork, **(D)** local property feature extraction and selection, **(E)** vector kernel, **(F)** subgraph feature extraction and selection, **(G)** graph kernel, **(H)** classification.

There were four parts to this study: data collection and preprocessing, construction of a high-order resting state hypernetwork for brain function, feature extraction and selection, and classification. Figure 1 shows a flowchart describing the entire process; specific aspects of this study are described below.

1. Data acquisition and preprocessing.
2. Construction of a high-order resting state hypernetwork for brain function.
  2.1. Group independent component analysis (GICA). The main steps of GICA included data dimension reduction, independent component estimation, data reconstruction, and noise elimination.
  2.2. Construction of a low-order functional brain network. Based on the average time series, time windows were divided using the time sliding window method. Based on each time window, the Pearson correlation method was used to obtain the connection matrix of the low-order functional brain network.
  2.3. Construction of a high-order resting state hypernetwork for brain function. We stacked the connection matrix of all low-order functional brain networks and then used the sparse group LASSO method to construct a brain functional hypernetwork.
3. Feature extraction and selection.
  3.1. We calculated the local topological properties of the brain functional hypernetwork as local property features. Then, we used the on-parametric permutation test to select features with significant differences.
  3.2. We extracted hyperedges as subgraph features. Then, the frequently FSFS method was applied to select discriminant subgraphs.
4. Construction of a classification model.
  4.1. The corresponding classifier was constructed by classification features that combined local property features and subgraph features.
  4.2. The cross-validation method was adopted to validate the classifier and obtain the final classification result.

### Data Acquisition and Preprocessing

Following the recommendations of the Shanxi Medical Ethics Committee (reference no. 2012013), all subjects needed to provide their consent to participate. All participants provided written informed consent in accordance with the Declaration of Helsinki, including 38 subjects with first-time, drug-free, major depression disorder (MDD) as the depression group and 28 age and gender-matched healthy volunteers as the normal control (NC) group. All subjects were righthanded. Participants in the depression group were first-time and drug-free patients identified by the criteria provided by the American Manual of Diagnostic and Statistical Manual of Mental Disorders, Fourth Edition (DSM-IV) (First and Gibbon, 1997). The severity of depression was determined by the 24 Hamilton rating scale for depression (HAMD) (Williams, 1988) and the clinical global impression of severity (CGI-S) (Guy, 1991). Using a 3T magnetic resonance scanner (Siemens Trio 3-Tesla scanner, Siemens, Erlangen, Germany), resting-state functional magnetic resonance scans were performed on 28 normal and 38 patients with depression. During the scan, subjects were requested to relax and their eyes closed, but not to fall asleep. Subjects wore spongy ear plugs and was placed carefully in the coil and provided cozy support. Detailed information relating to the subjects is shown in Table 1.

**TABLE 1:** Demographics and clinical characteristics of the study subjects.

	NC (*n* = 28)	MDD (*n* = 38)	*P*-value
Age (years)	17–51 (26.6 ± 9.35)	17–49 (28.4 ± 8.99)	0.41^a^
Gender (male/female)	13/15	15/23	0.55^b^
Handedness (R/L)	28/0	38/0	−
HAMD	NA	15–42 (22.8 ± 13.19)	−

*Data are presented as the range (mean ± SD). HAMD, Hamilton Depression Rating Scale; MDD, major depressive disorder. NA, not applicable; NC, normal control.*

*^a^P-value was calculated with the two-sample two-tailed t-test;*

*^b^P-value was computed by the two-tailed Pearson’s χ^2^ test.*

Data acquisition was completed by the First Hospital of Shanxi Medical University and all scans were performed by radiologists who were familiar with the operation of the MRI scanner. All patients underwent complete physical and neurological examinations, standard laboratory tests, and extensive neuropsychological assessments. During the scanning period, subjects were asked to close their eyes, relax, not to think about anything specific, but to remain awake and not to fall asleep. Scanning parameters were set as follows: 33 axial slices; repetition time (TR) = 2000 ms; echo time (TE) = 30 ms; slice thickness/skip = 4/0 mm; field of view (FOV) = 192 mm × 192 mm; matrix size = 64 mm × 64 mm; flip angle = 90°; volumes = 248.

Data preprocessing was performed in SPM8 software^1^. First, the dataset was corrected for slice time and head motion. From the final total of 66 subjects, data were not included from any subject with a head movement greater than 3 mm or with rotation greater than 3°. Then, we performed co-registration for spatial correction. Next, images underwent 12-dimensional optimal affine transformation into the standardized Montreal Neurological Institute (MNI) space, using 3 mm voxels. Smoothing was then performed to eliminate the differences between brain structures in different subjects and to improve the signal-to-noise ratio. Linear dimensionality reduction and bandpass filtering (0.01–0.10 Hz) were finally performed to eliminate the effects of line frequency drift and high frequency physiological noise. In addition, we used head, white matter, and cerebrospinal fluid signals as covariates for regression analysis to remove nuisance information from images. However, there was still disagreement in this field on whether global brain signals should be regressed, thus we did not regress global brain signals (Li et al., 2019).

### Group Independent Component Analysis

In the current study, GICA was used to analyze fMRI data. GICA was performed using the GIFT^2^ toolbox. Specifically, the minimum description length (MDL) criterion was adopted to estimate the optimal number of decomposition components (Koechlin and Summerfield, 2007) in the normal group and in the depression group. Based on this, the final number of independent components was set to 54. Next, each fMRI dataset was decomposed using the Infomax algorithm and 54 independent spatial components (ICs) were obtained. Please refer to Supplementary Text 1 and Supplementary Table 1 for a detailed explanation of the rationality for selecting 54 ICs. The core idea underlying the use of this algorithm was to minimize the mutual information among the components of the output by maximizing the mutual information between the input and the output (Du and Fan, 2013). In order to ensure the stability and reliability of the independent components, we ran the Infomax algorithm 20 times on ICASSO^3^ by randomly initializing the decomposition matrix; after these repetitions, the same convergence threshold (Nenert et al., 2014) was obtained. Finally, the GICA3 algorithm was used to reconstruct the data so that the spatial distribution and time series of the independent components of the subjects (Erhardt et al., 2011) could be obtained.

The ICs extracted by the GICA included not only the brain network components-of-interest in this paper but also other unrelated components and components with more noise. Therefore, it was necessary to use a previous template matching method to screen out these independent components and to further confirm the components-of-interest using a manual inspection method (Jafri et al., 2008). The screening criteria used for the exclusion of intrinsic connection network components included larger activation areas where the multiple regression coefficients matched the prior template; the distribution of the main activation regions in the gray matter, and the overlap of these regions with known components such as blood vessels and head movements in low frequency space; and the domination of the power spectrum of the time series in activation regions by low frequency power (Allen et al., 2011). Finally, 32 unrelated or noisy components were removed, and 22 brain network components were retained; these intrinsic connectivity network components were identified as being part of the auditory, sensorimotor, visual, default mode, attention, or frontal lobe networks.

### Construction of Resting State High-Order Functional Hypernetworks

Considering the dynamic changes of functional connections in the resting state, we proposed a high-order brain function hypernetwork model to simultaneously reflect the time-varying characteristics of the human brain’s working mechanism and the interactivity of multiple brain sections in space. Specifically, we first used the sliding time window method to construct a low-order functional brain network and then linked functional connections in multiple low-order functional brain networks into a relevant time series to reflect the time-variable characteristics of the functional connections. Based on the relevant time series, we used the sparse group LASSO method to construct a high-order functional hyper network. The specific steps are given below.

#### Construction of a Low-Order Resting State Functional Brain Network

Based on a fixed time window length and step size, the remaining 22 independent component time series data for each subject was divided into several time windows. The specific calculation for the number of sliding time windows is shown in Equation (1).


(1) W=⌊(T-l)/s+1⌋

In equation (1), *T* refers to the complete time series size of the fMRI data for each subject; *l* refers to the length of the sliding time window; *s* represents the step size of each sliding window, and *W* represents the number of time windows. Taking the *K*-th subject as an example, the time series is represented by *ts* ∈ ℝ^*T*×*N*^ and *N* represents the number of independent components. Using equation (1), *ts* ∈ ℝ^*T*×*N*^ can be divided into *W* overlapping sliding time windows, where each specific time window is represented by *ts*(*w*) ∈ ℝ^*l*×*N*^(1 ≤ *w* ≤ *W*), in other words, a rs-fMRI time series in a relatively short period of time.

Based on the time series for each sliding time windows *ts*(*w*) ∈ ℝ^*l*×*N*^(1 ≤ *w* ≤ *W*), we employed Pearson’s correlation method to obtain a functional connection network under each sliding time window; in other words, a low order resting state functional connection network. The specific calculation is shown in Equation (2).


(2) $$r_{i,j}=\frac{cov\left(i,j\right)}{\sigma_{i}\sigma_{j}}$$

In equation (2), *cov*(*i*, *j*) represents the covariance of the time series between the independent component *i* and the independent component *j*; σ_*i*_ represents the standard deviation of the time series of the independent component *i*; and *r*_*i*, *j*_ represents the correlation coefficient between the two components.

According to equation (2), we obtained *W* correlation matrices for each subject. In other words, we acquired *W* low-order resting state functional networks in a short period of time for each subject in which the nodes were independent components and the connection strength was *r*_*i*, *j*_. The *W* time window networks represented the time-varying characteristics of the brain functional connections over a short period of time. Considering the time-varying characteristics, the corresponding *r*_*i*, *j*_ in the *W* low-order functional connection networks could be linked into a relevant time series *TS* = [*r*_*i*, *j*_(1), *r*_*i*, *j*_(2),…, *r*_*i*, *j*_(*W*)]^*T*^ ∈ ℝ^*W*^ to reflect dynamic changes in functional connections. Note that the relevant time series *TS* = [*r*_*i*, *j*_(1), *r*_*i*, *j*_(2),…, *r*_*i*, *j*_(*W*)]^*T*^ ∈ ℝ^*W*^ has a different meaning from the time series for independent components *ts* ∈ ℝ^*T*×*N*^. The relevant time series *TS* = [*r*_*i*, *j*_(1), *r*_*i*, *j*_(2),…, *r*_*i*, *j*_(*W*)]^*T*^ ∈ ℝ^*W*^ represents the dynamic change in functional connections which mainly represents the time-variable characteristics of the functional connection. However, the latter *ts* ∈ ℝ^*T*×*N*^ represents the change of the specific independent component BOLD signal during the rs-fMRI scan.

Under this condition, after considering the rich time-variable characteristics, the corresponding relevant time series of data for each subject was represented by $CTS=\left[TS_{1},TS_{2},\ldots ,TS_{\frac{N\left(N-1\right)}{2}}\right]\in {\mathbb{R}}^{W\times \frac{N\left(N-1\right)}{2}}$. Of these, *TS*_1_ represents the relevant time series under the first functional connection; *W* represents the number of divided time windows; and $\frac{N\left(N-1\right)}{2}$ represents the number of functional connections in the low-order functional connection network, where *N* represents the number of independent components. In our study, $\frac{N\left(N-1\right)}{2}$ was 231, because *N* was 22.

#### Construction of a Resting State High-Order Functional Hypernetwork Based on the Sparse Group Least Absolute Shrinkage and Selection Operator Method

After identifying the changes in functional connectivity, we next constructed a brain functional hypernetwork. Here, we used the sparse group LASSO method to create a high-order functional hypernetwork for the resting brain (See Supplementary Text 2 for the reason that the sparse group lasso method was used to construct hypernetworks). The sparse group LASSO method is a bi-level and preset group selection method that can select variables at the group level, as well as individual variables within the group. In other words, groupwise and within-group variables can be freely selected, thereby filtering out some false connections while retaining some useful connections. In this manner, this method is a more effective means of characterizing the multiple and complex interactions in the human brain.

The sparse group LASSO method selects variables at the preset group level (Friedman et al., 2010a); therefore, before using this method to create a hypernetwork, the brain areas need to be grouped so that the brain areas with strong correlations are divided into one group. Then, the sparse group LASSO method was adopted to construct a brain function hypernetwork. Here, we used the k-medoids algorithm (Park and Jun, 2009) to carry out clustering. Specifically, according to the relevant time series data, the pairwise similarities between the functional connections were obtained; then, we performed clustering between the functional connections. When clustering, all functional connections were divided into *k* groups, where each group represented a class of objects; the relationship between objects and groups had to satisfy the following conditions: (1) each group implied at least one object, and (2) each object must belong to a group. To ensure the robustness of clustering, the principle of k-means++ (Benjamini and Hochberg, 1995) was used when selecting the initial clustering center in the clustering process. The specific process is as follows: (1) first, we set a cluster number *k* value and randomly selected a *k* point as the centroid; (2) we measured the distance between the remaining points and the selected *k* points, and then divided each remaining point into the nearest centroid cluster; (3) next, we reset the centroid and the new centroid was used to select the remaining points in which we used a random selection rule with a probability that was proportional to the distance of the data point from the nearest cluster center point; and (4) clustering was repeated 10 times, and the group with the best clustering effect during the period was selected as the final clustering result. After clustering, the sparse group LASSO method was introduced to construct the high-order function hyper network. The specific calculation is shown in Formula (3).


(3) $$\underset{\alpha_{m}}{min}{||TS_{m}-CTS_{m}\alpha_{m}||}_{2}+\lambda_{1}{||\alpha_{m}||}_{1}+\lambda_{2}\sum_{i=1}^{k}{||\alpha_{mG_{i}}||}_{2}$$

*TS*_*m*_ represents the relevant time series of the *m*-th functional connection. *CTS*_*m*_ = [*TS*_1_,…, *TS*_*m*−1_,0, *TS*_*m* + 1_,…, *TS*_*M*_] represents the data matrix of the *m*-th functional connection (all relevant time series except for the *m*-th functional connection, that is, the relevant time series corresponding to the *m*-th functional connection was set to 0), where $M=\frac{N\left(N-1\right)}{2}$. α_*m*_ represents the weight vector, which quantifies the degree of influence from other functional connections on the *m*-th functional connection. This is divided into k non-overlapping tree groups α_*mG*_1__,α_*mG*_2__,…,α_*mG*_*k*__ through clustering, where *G*_*i*_(*i* = 1,2,…, *k*) represented a node with a tree structure. The functional connections corresponding to the non-zero element in α_*m*_ represented the functional connections that interact with the specific functional connection *TS*_*m*_. Conversely, the zero element indicates that the corresponding functional connection was independent of the *m*-th functional connection, and no interaction existed.λ_1_,λ_2_ represented regularization parameters: λ_1_ was used to adjust the sparsity in the group, that is, to control the number of non-zero coefficients in the non-zero group. If λ_1_ was different, then the sparsity within the group was different; in other words, the number of non-zero coefficients in the group were different. λ_2_ was used to adjust the group-level sparsity (Yuan and Lin, 2006; Friedman et al., 2010b) and control the number of groups with at least one non-zero coefficient. If λ_2_ was different, then the group-level sparsity was different; in other words, the selected group variables were different. In the experiment, we solved the optimization problem by applying the sparse group LASSO method in the SLEP package (Liu et al., 2013).

**FIGURE 2:**
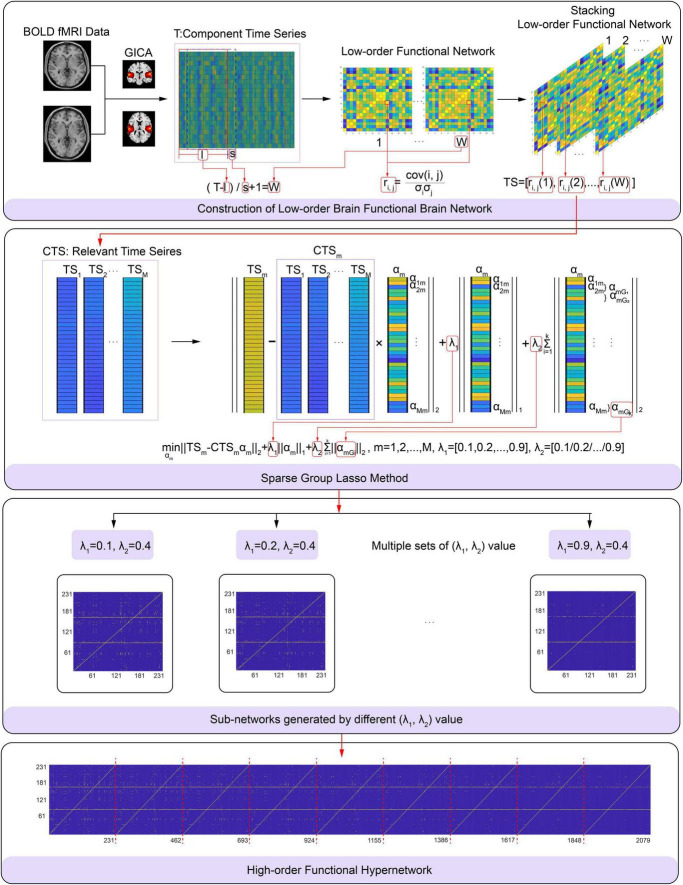
Flowchart showing the construction of a high-order brain functional hyper-network based on the sparse group LASSO method, including **(A)** construction of low-order brain functional networks, **(B)** schematic diagram of the sparse group LASSO method, **(C)** sub-networks generated by differentλ_1_,λ_2_), **(D)** high-order brain functional hyper-network.

Specifically, in each subject, α_*m*_ was measured using formula (3) based on the relevant time series and considering multi-level neural activity information for a selected function connection; we did this by fixing the λ_2_ value and varying the λ_1_ value from 0.1 to 0.9 with a step size is 0.1. The functional connection corresponding to non-zero elements in α_*m*_ and a selected function connection consists of a hyperedge. All hyperedges formed a high-order hypernetwork. The process used to construct a specific high-order brain function hypernetwork is shown in Figure 2. In this experiment, we set λ_2_ to 0.4 because this achieved the highest level of accuracy compared with other λ_2_ values.

### Feature Extraction and Selection

In current study, we introduced a new property, shortest path length (Zhang and Liu, 2010), into our neuroimaging research and combined this with the traditional clustering coefficient to evaluate local topology information in the high-order functional hypernetwork from multiple angles. In addition, considering that the local topological properties could not fully characterize the topological information of the hypernetwork, we introduced hyperedges as subgraph features to characterize global topological information of the high-order brain function hypernetwork. The specific definition for local topological properties and global properties is described below.

#### Local Property Feature Extraction and Selection

We introduced multiple different types of topological attributes to the high-order hypernetwork from different angles, including the clustering coefficient and the shortest path distance. The clustering coefficient included three hypernetwork clustering coefficients based on a single node (HCC) and a hypernetwork clustering coefficient based on a pair of nodes (HCCPN). The specific definition is described below.

The first type of clustering coefficient based on a single node represents the number of adjacent nodes that have connections not included by node *v* and is represented by HCC^1^. The advantage of this definition is that any interaction found in this set represents the real connection between neighboring nodes and that there will be no data artifacts caused by interaction with node *v* (Gallagher and Goldberg, 2013). However, this definition may focus too much on neighbors with secondary shared connections that have nothing to do with node *v*. The calculation for this is shown in Equation (4):


(4) $$HCC^{1}\left(v\right)=\frac{2\sum_{u,t\in N\left(v\right)}I\left(u,t,\neg v\right)}{\left|N\left(v\right)\right|\left(\left|N\left(v\right)-1\right|\right)}$$

The *u*, *v*, *t* represent nodes. *N*(*v*) = {*u* ∈ *V*:∃*e* ∈ ζ, *u*, *v* ∈ *e*} represent the neighbors of node *v*, where ζ represents the set of hyperedges; *e* represents a hyperedge. If∃*e* ∈ ζ, when *u*, *t* ∈ *e* and *v*∉*e*, *I*(*u*, *t*,¬*v*) = 1. Otherwise, *I*(*u*, *t*,¬*v*) = 0.

The second type of clustering coefficient based on a single node represents the ratio of adjacent nodes containing node *v* that are also adjacent to each other and represented by HCC^2^. The advantage of this definition is that it is more likely to find the true connection between node *v* and the neighboring nodes. However, inevitably, the interactions discovered in this way may include data artifacts due to the shared interaction with node *v* (Gallagher and Goldberg, 2013). The calculation for this is shown in Equation (5).


(5) $$HCC^{2}\left(v\right)=\frac{2\sum_{u,t\in N\left(v\right)}I^{\prime }\left(u,t,v\right)}{\left|N\left(v\right)\right|\left(\left|N\left(v\right)-1\right|\right)}$$

The *u*, *v*, *t, N*(*v*) have the same meaning as the parameters in HCC^1^. If ∃*e* ∈ ζ, when *v*, *u*, *t* ∈ *e*, *I*′(*u*, *t*, *v*) = 1. Otherwise, *I*′(*u*, *t*, *v*) = 0.

The third type of clustering coefficient based on a single node is the overlap ratio of adjacent hyperedges of node *v* and is represented by HCC^3^ (Gallagher and Goldberg, 2013). This definition represents the ratio of shared edges between node *v* and its neighbor nodes. The calculation for this is shown in Equation (6).


(6) $$HCC^{3}\left(v\right)=\frac{2\sum_{e\in S\left(v\right)}\left(\left|e\right|-1\right)-\left|N\left(v\right)\right|}{\left|N\left(v\right)\right|\left(\left|S\left(v\right)-1\right|\right)}$$

The *v, e, N*(*v*) also have the same meaning as the parameters in HCC^1^. *S*(*v*) = {*e* ∈ ζ:*v* ∈ *e*} represents hyperedges containing node *v*.

The first type of clustering coefficient based on pairs of nodes represents a compromise between meeting the maximum and minimum criteria and is represented by HCCPN^1^. Of these, the maximum criterion considers that there may be obvious overlap between neighbors, defined by$\frac{\left|S\left(u\right)⋂S\left(v\right)\right|}{\max\left\{|S\left(u\right),S\left(v\right)|\right\}}$. The minimum criterion considers the fact that a small neighborhood may intersect with a large neighborhood, defined by $\frac{\left|S\left(u\right)⋂S\left(v\right)\right|}{\min\left\{|S\left(u\right),S\left(v\right)|\right\}}$ (Gallagher and Goldberg, 2013). The calculation for this is shown in Equation (7).


(7) $$HCCPN_{(}^{1}u,v)=\frac{\left|S\left(u\right)⋂S\left(v\right)\right|}{\sqrt{\left|S\left(u\right)\right|\left|S\left(v\right)\right|}}$$

*v and u* represent nodes and *S*(*v*) has the same meaning as the parameters in HCC^3^.

After calculating the clustering coefficient based on pairs of nodes, the specific clustering coefficient of the node was obtained by averaging the clustering coefficients of the node and all its neighbor nodes (Latapy et al., 2008). The calculation for this is shown in Equation (8).


(8) $$HCCPN\left(v\right)=\frac{\sum_{u\in N\left(v\right)}HCCPN^{1}\left(u,v\right)}{\left|N\left(v\right)\right|}$$

HCCPN^1^(*u*, *v*) represents the clustering coefficient based on pairs of nodes. *N*(*v*) has the same meaning as the parameters in HCC^1^.

The shortest path length represents the shortest distance from the selected node to all other nodes (Zhang and Liu, 2010). In the hypernetwork, the path was defined according to the hyperedges from the source node to each destination node; the length of the path depended on the number of hyperedges along the path. If the hypergraphs are weighted hypergraphs, then, the weights of the hyperedges also need to be considered. If the hypergraphs are binary undirected hypergraphs, then the shortest path is the mean value of the minimum number of hyperedges passing through the path from the source node to each destination node. This indicator is often used in social and protein networks (Zhang and Liu, 2010). The calculation is shown in Equation (9).


(9) $$SP\left(v\right)=\frac{\sum_{v\in V,v\neq u}d\left(u,v\right)}{V-1}$$

SP(*v*) represents the shortest path of node *v* in the hypergraph *H* while *d*(*u*, *v*) represents the shortest path between nodes *u* and *v*. *V* represents the number of nodes in the hypernetwork.

These five local topological properties comprehensively reflected the local topological information of the high-order hypernetwork. Then, based on each topological attribute, we adopted multiple linear regression analysis to estimate the influence of confounding factors (demographic information) on the local properties of the hypernetwork. Specifically, for each participant, each local topological attribute was averaged and identified as an independent variable (average HCC^1^, average HCC^2^, average HCC^3^, average HCCPN, and average SP), and demographic information was identified as the dependent variable. Then multiple linear regression was performed. See Supplementary Text 3 for the results. The results show that there is no significant correlation between all local topological attributes and confounding variables, that is, they are not affected by confounding factors.

Features extracted from a high-order hypernetwork may contain some irrelevant or redundant features. Therefore, to select key features for classification, the most discriminative features were selected based on statistical differences. For the MDD and NC group, we used the Kolmogorov–Smirnov non-parametric permutation test (KS non-parametric permutation test) (Fasano and Franceschini, 1987) for 1155 properties extracted from local properties. This data was then corrected by the Benjamini–Hochberg false-discovery rate (FDR) method (*q* = 0.05) (Benjamini and Hochberg, 1995). Following the KS non-parametric permutation test, the local attributes showing significant differences between groups were used as classification features (vector kernel). These were then fused by multi-kernel learning to construct the classification model. Note that the concatenation method was applied to combine the difference features of multiple local attributes.

#### Subgraph Feature Extraction and Selection

Previous studies have shown that subgraph features can express global attributes in brain networks and have been effectively used for the diagnosis of brain disease (Kong et al., 2013; Guo et al., 2018b). Therefore, we introduced subgraph features to describe the global information of the high-order brain hypernetwork. In the hyper-network, the hyperedges could be regarded as subgraphs. Therefore, we directly extracted hyperedges from the high-order brain functional hypernetwork as subgraph features.

The number of subgraphs extracted by hyperedges was very large. If all subgraphs participated in the classification, then the classification performance would be reduced. This is because not all frequent subgraphs have discriminative ability; in fact, only a few subgraphs show discriminative ability (Guo et al., 2017). Thus, it was necessary to select discriminative subgraphs as classification features. Here, we adopted the frequent scoring feature selection (FSFS) method to select discriminant subgraphs. Specifically, the discriminative scores (i.e., the frequency difference) of subgraph features were, respectively, calculated and sorted into two groups. Then, the features with larger frequency differences between the two groups of subjects were extracted as discriminative subgraphs.

The specific concepts and symbols used in the FSFS method are explained in the following formulae.


(10) $$D:D=\left\{D_{n},D_{p}\right\}.$$

In Formula (10), *D*_*n*_ represents a negative sample (patients with depression) while *D*_*p*_ represents a positive sample (normal control).


(11) $$\varsigma :\varsigma =\left\{\varsigma_{n},\varsigma_{p}\right\}.$$

In Formula (11), ς_*n*_ = {*g*_*n*1_, *g*_*n*2_,…, *g*_*nk*_} represents the feature set of all subgraphs in the negative sample; this represents the set of all hyperedges in patients with depression. In contrast, ς_*p*_ = {*g*_*p*1_, *g*_*p*2_,…, *g*_*pk*_} represents the feature set of all subgraphs in the positive sample; this represents the set of all hyperedges in normal subjects.


(12) $$\Omega^{*}=\Omega_{1}^{*}⋃\Omega_{2}^{*}.$$

In Formula (12), $\Omega_{1}^{*}\subseteq \varsigma_{p}$; $\Omega_{2}^{*}\subseteq \varsigma_{n}$; Ω*⊆ς represents the optimal set of subgraph features, as determined by Formula (13).


(13) $$\Omega^{*}=\underset{\Omega_{1}\subseteq \varsigma_{p},\Omega_{2}\subseteq \varsigma_{n}}{argmax}J\left(\Omega \right)s.t\left|\Omega_{1}\right|\leq maxt_{1},\left|\Omega_{2}\right|\leq maxt_{2}$$

In Formula (13), |⋅| represents the number of subgraph feature sets while *maxt*1, *maxt*2 represent the maximum number of subgraph features selected in the two groups of subjects, respectively. *J*(Ω) represents the validity criterion for evaluating the feature subset of the subgraph, and was calculated by Formula (14) and Formula (15).


(14) $$J\left(\Omega \right)=\sum_{i\leq t_{1}}S\left(g_{pi}\right)+\sum_{j\leq t_{2}}S\left(g_{nj}\right)$$

(15) $$S\left(g_{s}\right)=|f_{q}\left(g_{s}|D_{p}\right)-f_{q}\left(g_{s}|D_{n}\right))|$$

*S*(*g*_*s*_) represents the discriminative score of the subgraph pattern *g*_*s*_, expressed by the frequency difference between a positive sample and a negative sample. The greater the frequency difference, the stronger the discriminative ability of the subgraph feature between the two groups of subjects. An *S*(*g*_*s*_) = 1 implies that the subgraph pattern *g*_*s*_ only exists in only one group of subjects. That is, the subgraph pattern only appears in the normal control group or only in the depression group. The discriminant scores of subgraphs in the two groups of subjects were, respectively, calculated using Formula (15) and sorted using Formula (16).


(16) $$S\left(g_{p}^{1}\right)\geq S\left(g_{p}^{2}\right)\ldots \geq S\left(g_{p}^{m}\right),S\left(g_{n}^{1}\right)\geq S\left(g_{n}^{2}\right)\ldots \geq S\left(g_{n}^{k}\right)$$

By applying Formula (16), the optimal subgraph feature set was obtained as shown in Formula (17).


(17) $$\Omega^{*}=\left\{g_{p}^{i},g_{n}^{j}|i\leq t_{1},j\leq t_{2}\right\}$$

In Formula (17), $g_{p}^{i}$ represents the *i*-th discriminant subgraph in a positive sample and $g_{n}^{j}$ represents the *j*-th discriminant subgraph in a negative sample. *t*_1_ represents the number of discriminant subgraphs in a positive sample while *t*_2_ represents the number of discriminant subgraphs in a negative sample.

According to the FSFS method, the discriminant subgraphs from the two groups of subjects were selected. Because the subgraphs could not be directly used as classification features to participate in the construction of the SVM classification model; first, they needed to be quantified. Thus, based on the discrimination subgraphs, we adopted the graph kernel method (Shervashidze et al., 2011) to quantify data into a graph kernel matrix.

Research shows that the graph kernel model is a commonly used strategy for isomorphic subgraph testing and serves as a link between graph data and many machine learning methods. In other words, the graph can be converted from the original space to the vector space; then, the similarity can be calculated (test graph isomorphism). Over recent years, researchers have introduced a variety of graph kernel measurement methods, such as subtree-based kernels (Shervashidze et al., 2011), path-based methods (Alvarez et al., 2011) and walk-based methods (Gärtner et al., 2003). In neuroimaging, the Weisfeiler-Lehman subtree kernel was proven to effectively capture topological information from graphs and achieved better performance than other graph kernel methods (Shervashidze et al., 2011). Therefore, we introduced the Weisfeiler-Lehman subtree kernel method to quantify discriminant subgraphs (Shervashidze et al., 2011). Here, an iterative method was used to relabel the original node label. In each subsequent iteration, the label for each node was replaced based on the label obtained in the last iteration and the label of its neighboring nodes. This continued until the labels of all nodes were the same, or the number of iterations was a predefined maximum value. For the specific construction process of the Weisfeiler-Lehman subtree kernel, see Supplementary Text 4. In current study, based on a discriminative subgraph$g_{p}^{i}$, we combined each subject’s high-order hypernetwork separately to perform the Weisfeiler-Lehman test of isomorphism. The obtained value was used as the graph kernel feature of the subgraph $g_{p}^{i}$. Similarly, the graph kernel features of all discriminative subgraphs were calculated. Finally, all graph kernel features were formed into graph kernel matrix to participate in the construction of the classification model.

### Classification

Based on the two types of features, we introduced multi-kernel learning to merge the vector kernel and the graph kernel into a mixed kernel, thus providing complementary information to improve the construction of the classification model. Specifically, the kernel-based feature combination was used to estimate the different weights of each group of features for feature fusion. This allowed multiple kernels functions to be merged into a hybrid kernel to participate in the construction of the classification model. The specific function of the hybrid kernel is shown by Equation (18).


(18) $$k_{f}\left(x,z\right)=\sum_{l=1}^{L}\mu_{l}k_{f}^{l}\left(x,z\right)$$

In Equation (18), $k_{f}^{l}\left(x,z\right)$ represents the kernel function of the *l*-th group of features (the *l*-th group of topological attributes) between subject *x* and subject *z*. μ = {μ_1_,μ_2_…,μ_*L*_} represents the combined parameters of the kernel matrix and ∥μ∥_2_ = 1, where *L* represents the number of kernel matrices (in the experiment, *L* = 2). *k*_*f*_(*x*, *z*) represents a mixed kernel.

In multi-kernel learning, the most critical step is to determine the combination parameter μ. This directly affects the data fusion method and ultimately affects the classification performance. Here, we used the alignment maximization algorithm to determine the weight of the parameter μ (*l* = 1,…, *L*) (Cortes et al., 2010). This algorithm mainly seeks to maximize the alignment between the basic kernel *k*f** and the target kernel *k*y** to determine μ. The optimization function is shown in Formula (19).


(19) $$\max_{\mu \in L}\frac{<k_{f},k_{y}{>}_{F}}{{||k_{f}||}_{F}}$$

In Formula (19), *k*y* = yy*^T^**, *y* is a label. <.,.>*_*F*_* represents the Frobenius inner product, ∥.∥*_*F*_* represents the Frobenius norm. To solve Formula (19), let *b* express the vector $\left(<k_{f}^{1},yy^{T}{>}_{F},\ldots ,<k_{f}^{L},yy^{T}{>}_{F}\right)$ and ***F*** represent the matrix characterized by $F_{mn}=<k_{f}^{m},k_{f}^{n}{>}_{F}$ (*m*, *n* = 1…*L*). Formula (19) is transformed into a quadratic programming problem, expressed as Equation (20).


(20) $$\mu =\frac{F^{-1}b}{||F^{-1}b||}$$

After obtaining the value of μ, multiple kernels were fused to a hybrid kernel, so that we could construct a classification model using the traditional SVM classifier based on the libsvm package^4^.

We adopted ‘leave one out cross validation’ (LOOCV) to evaluate classification performance. If there were N samples, each sample was, respectively, regarded as the test set, and the remaining N-1 samples were regarded as the training set. Then, in the training set, K-fold Cross Validation was used for parameter optimization (c, γ) and the parameter group (c, γ) with the highest classification accuracy in the training set was selected to construct the classification model (Mishra and Deepthi, 2020). Here, we set the range of (*c*, γ) to (2^–7^, 2^7^). In this way, a total of *N* different classification models was established. Next, the test set was used to predict the model. Note that before the classification model was constructed, the classification features needed to be standardized. In addition, considering the influence of the random selection of initial random seed points of the clustering algorithm during the construction of the high-order function hyper network, we repeated 50 experiments. The average value of the 50 experiments was considered as the final classification result.

## Results

### The Intrinsic Connectivity Network

**FIGURE 3:**
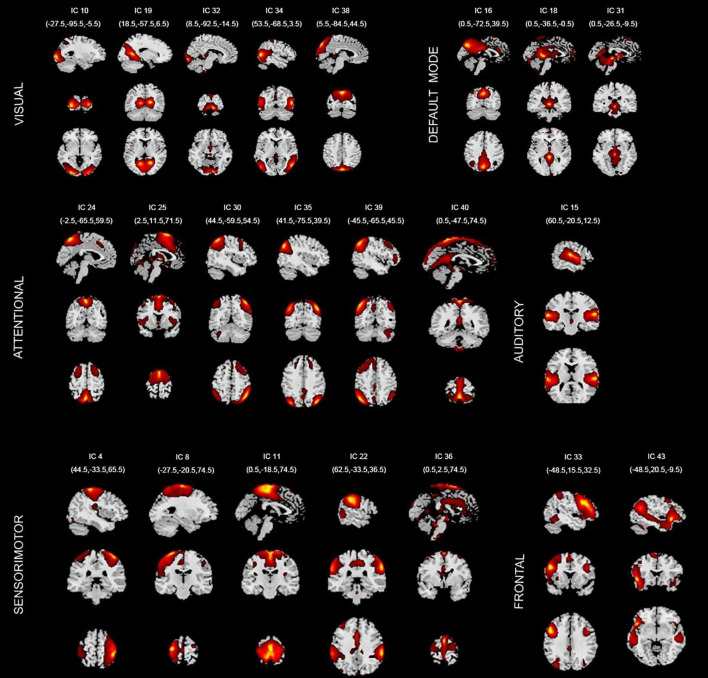
Spatial maps of the 22 components identified as intrinsic connection network (ICNs). VISUAL, visual network; DEFAULT MODE, default mode network; ATTENTIONAL, attentional network; AUDITORY, auditory network; SENSORIMOTOR, sensorimotor network; FRONTAL, frontal network. IC, independent component.

In this paper, 22 independent components were selected from the GICA. Figure 3 shows the spatial maps of these 22 independent components. According to the spatial maps of each independent component, the inherently connected network to which they belong was determined, as shown in Figure 3. In addition, we supplemented the coordinates of peak activations corresponding to each of these components, as shown in Supplementary Table 2 below.

These 22 ICs were similar to those identified in previous work (Beckmann et al., 2005; Calhoun et al., 2008; Smith et al., 2009; Allen et al., 2011). Here, we described these 22 ICs in detail and provided citations to more comprehensive references. Resting-state networks are grouped by their anatomical and functional properties. IC 15 forms a rather prototypical representation of the large parts of the auditory system (AUD), mainly including bilateral activation of the superior temporal gyrus. Seifritz et al. (2002) indicated the temporal lobe was associated with the auditory system. Specht and Reul (2003) found a functional segregation of the temporal lobes into several subsystems responsible for auditory processing was visible.

The Sensorimotor networks (SM) were captured by five components (ICs 4, 8,11,22, and 36) situated in the vicinity of the central sulcus, mainly including activation of the left precentral gyrus, right postcentral gyrus, bilateral activation of the paracentral lobule, supramarginal gyrus and supplementary motor area. Parkinson et al. (2012) examined the fMRI correlates of speech feedback processing during an active speaking task with and without pitch perturbation in the auditory feedback. Results revealed a complex sensory–motor network involved in speech feedback processing including precentral gyrus, postcentral gyrus, supplementary motor area, etc. Hänggi et al. (2017) highlighted the neurological underpinnings of Xenomelia by assessing structural and functional connectivity by means of whole-brain connectome and network analyses of regions involved in Xenomelia. They illustrated subnetworks showing structural and functional hyperconnectivity in xenomelia compared with controls. These subnetworks were lateralized to the right hemisphere and mainly comprised by nodes belonging to the sensorimotor system, including the paracentral lobule, supplementary motor area, postcentral gyrus, etc. Agcaoglu et al. (2015) evaluated resting state network lateralization in an age and gender-balanced in functional magnetic resonance imaging (fMRI) dataset. The result showed that age was strongly related to lateralization in multiple regions including sensorimotor network regions precentral gyrus, postcentral gyrus and supramarginal gyrus.

The visual system (VIS) is also represented by six components (ICs 10, 19, 32, 34, and 38) in good agreement with the anatomical and functional delineations of occipital cortex. The main active regions were the lingual gyrus, cuneiform lobe, suboccipital gyrus, talus gyrus and middle temporal gyrus. Agcaoglu et al. (2015) showed the visual network was the most dominantly right lateralized functional network, including lingual gyrus, talus gyrus, cerebellum, suboccipital gyrus and inferior temporal gyrus. Ten Donkelaar and Cruysberg (2020) showed that lesions of the cuneus and posterior parietal lobe cause visuospatial disorders such as impaired motion perception, spatial disorientation and defects in attention. In addition, many researches have proved that the visual cortex evolved a region in the middle-temporal cortex that is highly specialized to process visual motion (Zeki, 1974; Zeki and Whitteridge, 1980; Baker et al., 1981).

The default mode network (DMN) was captured by three independent components (ICs 16, 18, and 31); the main active regions were located in the precuneus lobe, lingual gyrus and temporal lobe, etc. Cunningham et al. (2017) demonstrated that a detailed mapping of connectivity between the precuneus and thalamus and their connectivity with the DMN would provide a comprehensive baseline for future brain imaging studies, especially those involving consciousness. Dalwani et al. (2014) investigated whether DMN was altered in adolescents with conduct disorder and substance use disorders, relative to controls. The result showed that compared to controls, patients indicated reduced activity in superior, medial and middle frontal gyrus, retrosplenial cortex and lingual gyrus, and bilateral middle temporal gyrus—DMN regions thought to support self-referential evaluation, memory, foresight, and perspective taking.

The attention network (ATTN) was captured by six independent components (ICs 24, 25, 30, 35, 39, and 40); the main active regions were located in the frontal lobe, parietal lobe, precuneus lobe, temporal lobe and angular gyrus. Allen et al. (2011) classified several ICs known to be involved in directing and monitoring behavior as attentional networks. These included lateralized frontal-parietal networks (IC 30 and 39) similar to the ventral attention network. Some studies showed that precuneus lobe (IC 24) was implicated in directing attention (Cavanna and Trimble, 2006; Margulies et al., 2009). Agcaoglu et al. (2015) indicated that age was strongly related to lateralization in multiple regions with inferior parietal lobule, superior parietal lobule and middle temporal gyrus in attention network.

Finally, frontal networks (FRONT; ICs 33 and 43) known to mediate executive as well as memory and language functions was observed, whose active regions were located in the medial prefrontal cortex and parietal lobe (Koechlin et al., 2003; Koechlin and Summerfield, 2007). Therefore, it can be seen that the regions activated by independent components are consistent with previous findings.

### Abnormal Components Based on Local Properties

**FIGURE 4:**
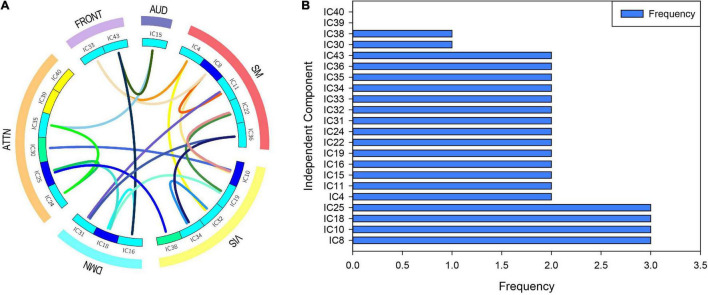
Abnormal independent components of the local attribute features. AUD, auditory network; SM, sensorimotor network; VIS, visual network; DMN, default mode network; ATTN, attentional network; FRONT, frontal network. IC, independent component. **(A)** The independent components involved in the 21 differential functional connections. Edges of different colors indicate different functional connections. Nodes of different colors indicate the frequency of the component in all abnormal functional connections. **(B)** The number of occurrences for each independent component in the abnormal functional connection.

Once the resting-state high-order function hypernetwork had been constructed, the local topological attributes were used to quantify the network to obtain local attribute features. The KS non-parametric test was then used to obtain local features with significant differences (with appropriate FDR correction). These significant features represented functional connections between components. The statistical significance results for specific differential functional connection are shown in Table 2; there were 21 abnormal functional connections. The independent components involved in the 21 differential functional connections are also shown in Figure 4A, including 20 components. Furthermore, we counted the number of occurrences for each independent component in the abnormal functional connection, as shown in Figure 4B. The results showed that the top four abnormal independent components with the highest frequencies were IC8, IC10, IC18, and IC25 (a total of three times). This showed that among all independent components, these independent components were the most discriminative. The corresponding inherent connection networks were the sensory motor, visual, default mode and attention networks.

**TABLE 2:** Discriminative functional connectivity based on local attributes.

Functional connection		Local topological properties (*P*-value)				
IC A	IC B	HCC^1^	HCC^2^	HCC^3^	SP	HCCPN
4	32	**0.007**	**0.022**	**0.028**	**0.003**	0.756
4	33	**0.002**	**0.033**	**0.004**	**0.005**	0.891
8	10	**0.022**	**0.003**	**0.026**	0.691	0.497
8	11	**0.002**	**0.007**	**0.002**	0.415	0.485
8	33	**0.000**	**0.004**	**0.000**	**0.004**	0.850
10	22	0.094	**0.008**	**0.002**	0.074	0.781
10	30	**0.049**	**0.004**	**0.014**	0.163	0.485
11	31	**0.001**	**0.011**	**0.004**	0.277	0.415
15	35	**0.001**	**0.033**	**0.026**	0.963	**0.011**
15	43	0.094	0.691	0.188	0.891	**0.001**
16	18	**0.012**	0.303	0.372	0.285	0.229
16	43	0.828	**0.038**	0.137	0.114	0.625
18	19	0.461	0.625	0.839	0.730	**0.010**
18	25	**0.040**	**0.017**	**0.022**	0.074	0.963
19	22	**0.021**	**0.007**	**0.003**	**0.000**	0.382
24	25	0.963	0.665	0.691	0.426	**0.003**
24	35	0.142	0.473	0.871	0.891	**0.022**
25	38	0.294	0.438	0.094	0.817	**0.000**
31	36	**0.001**	**0.003**	**0.038**	0.147	0.106
32	34	**0.016**	0.322	**0.007**	**0.005**	0.891
34	36	0.268	**0.003**	**0.043**	0.449	0.510

*ICA and ICB represent two independent components in functional connection; HCC^1^ represents the first type of hypernetwork clustering coefficient based on a single node; HCC^2^ represents the second type of hypernetwork clustering coefficient based on a single node; HCC^3^ represents the third type of hypernetwork clustering coefficient based on a single node; SP represents the shortest path; HCCPN represents the hypernetwork clustering coefficient based on pairs of nodes. The bold values represents P < 0.05.*

### Discriminative Components Based on Subgraph Features

**FIGURE 5:**
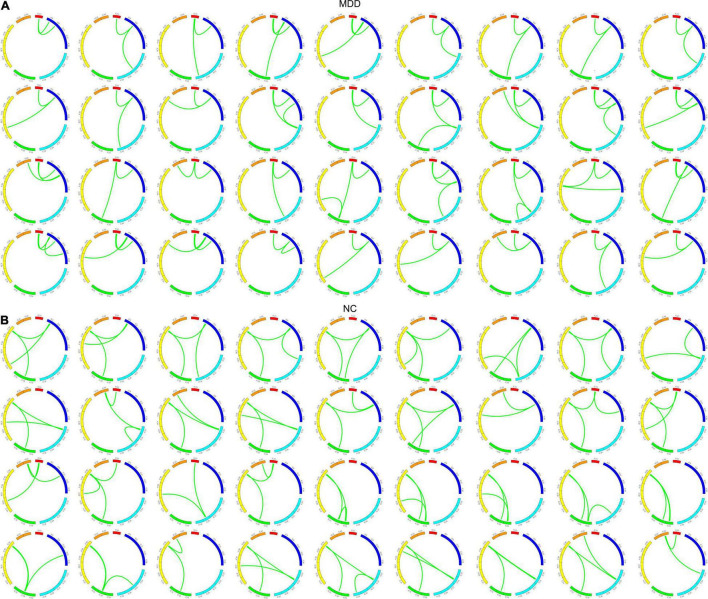
Discriminant subgraphs in the two groups of subjects. **(A)** The top 36 discriminative subgraphs in the MDD group, **(B)** the top 36 discriminative subgraphs in the NC group.

**FIGURE 6:**
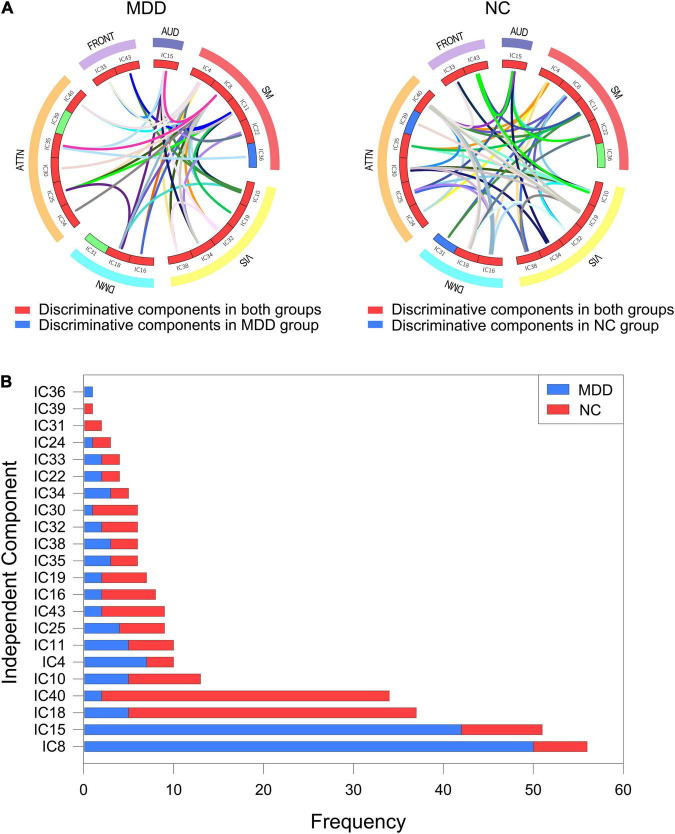
Discriminative components of the discriminate subgraph features. AUD, auditory network; SM, sensorimotor network; VIS, visual network; DMN, default mode network; ATTN, attentional network; FRONT, frontal network. IC, independent component. **(A)** All discriminative subgraphs were combined in each group. Left figure represents all discriminative components of the discriminate subgraph features in MDD group. Right figure represents all discriminative components of the discriminate subgraph features in NC group. **(B)** A statistical chart showing the frequencies of the independent components in two groups of subjects. Red represents MDD group; Blue represents NC group.

Based on the resting state high-order brain function hypernetwork model, except for using quantifiable indicators to calculate local attribute features and we also extracted hyperedges as subgraph features to characterize global topological information. And we adopted FSFS methods to select discriminate subgraphs. Here, to ensure a balanced number of subgraph features, we, respectively, selected the top 36 frequent subgraphs with the highest frequency difference in the two groups of subjects as the discriminative subgraph features to perform classification, which was shown in Figure 5 (see the Supplementary Text 5 for a relative discussion of the number of discriminative subgraph features). To easily analyze the difference between the subgraph features for the two groups, we combined all discriminant subgraphs obtained for each group of subjects, as shown in Figure 6A. The results showed that the discriminative components obtained by the two sets of discriminative subgraphs were mostly the same. However, there are significant differences in these common components, namely IC8, IC15, IC18, IC40, IC10, IC4, IC11, IC25, IC43, IC16, IC19, IC35, IC38, IC32, IC30, IC34, IC22, IC33, and IC24. On this basis, we counted the number of times each independent component appeared in all discriminative subgraphs to select the most discriminative components on MDD, as shown in Figure 6B. The results showed that the top four discriminative components were IC8, IC15, IC18, and IC40. Of these, IC8 had the largest number of occurrences in discriminative components (56 times); this was followed by IC15 (51 times), IC18 (37 times), and IC40 (34 times). This showed that for subgraph features, these independent components were the most discriminative. The corresponding inherent connection networks were the sensory motor, auditory, default mode and attention networks.

### Classification Results

We evaluated the classification performance of the RS-HOFHN model by classification accuracy, sensitivity, specificity, balanced accuracy (BAC), and area under curve (ROC). First, we separately calculated the classification accuracy of a single feature (local attribute feature and subgraph feature) and the classification accuracy of the fusion feature under the proposed method. Next, using the same data set, we compared the classification performance of the rs-HOFHN model with a traditional simple binary functional network (TBFN), a resting state high-order functional network (rs-HOFN), and a resting state functional hypernetwork (rs-FHN) model. For the TBFN model, Pearson correlation was first used to construct a functional brain network. Then, local attribute features were obtained by calculating the degree, betweenness centrality, and node efficiency; local difference features were then selected using KS non-parametric tests. Next, the gSpan algorithm (Yan and Han, 2002) was introduced to calculate frequent subgraphs. The FSFS algorithm was then applied to obtain the discriminant subgraphs. Finally, we merged the two sets of features for SVM classification. For the rs-HOFN model, the sliding window method was first used to construct the high-order function connection network. Feature extraction, selection, and classification processes in the rs-HOFN model were the same as for the TBFN. We used the sparse group LASSO method to construct the brain function hypernetwork for the rs-FHN model. Then, the five local attributes selected in this experiment were introduced to acquire local features and KS non-parametric tests were employed to select local difference features. Next, hyperedges were set as subgraph features, and the FSFS algorithm was also used to obtain the discriminant subgraphs. Finally, the two types of features were merged to construct the SVM classifier. The classification results are shown in Table 3. The results show that the classification accuracy of the rs-HOFHN model was the highest; the fusion feature reached 92.18%, which was superior to the classification performance of the TBFN, rs-HOFN and HFN models. In addition, except that the sensitivity of fusion features was slightly lower than that of local properties, the rest of the classification results show that the fusion features were better than single features in each network model. Furthermore, we compared the classification performance obtained by the rs-HOFHN model with the rest of the hypernetwork models in the existing researches (the hypernetwork model constructed by the star expansion method, the LASSO method, the elastic net method, and the group LASSO method), as shown in Table 3. The results show that the rs-HOFHN model proposed in this paper achieved the best classification performance, outperforming the existing hypernetwork models.

**TABLE 3:** Comparison of classification results for different methods.

Method	Research	Accuracy (%)	Sensitivity (%)	Specificity (%)	BAC (%)
TBFN	Local properties	74.24	78.95	67.86	73.41
	Subgraph feature	72.72	76.32	67.86	72.09
	Fusion feature	78.79	81.57	75.00	78.29
rs-HOFN	Local properties	84.84	89.47	78.57	84.02
	Subgraph feature	81.82	86.84	75.00	80.92
	Fusion feature	89.39	92.11	85.71	88.91
rs-FHN	Local properties	86.42	89.00	82.92	85.96
	Subgraph feature	80.30	82.42	77.00	79.71
	Fusion feature	89.18	90.95	86.77	88.86
rs-HOFHN	Local properties	89.55	93.95	83.57	88.76
	Subgraph feature	84.69	87.05	81.48	84.27
	Fusion feature	92.18	93.63	90.20	91.92
Star expansion	Yang et al. (Li et al., 2017)	74.10	76.50	70.00	73.20
LASSO	Yang et al. (Li et al., 2019)	75.40	64.30	84.90	74.60
Elastic net	Guo et al. (2018a)	86.36	92.10	81.57	86.83
Group LASSO	Li et al. (2020)	81.74	84.74	77.68	81.21

*TBFN represents traditional simple binary functional network model; rs-HOFN represents resting state high-order functional network; rs-FHN represents resting state functional hypernetwork; rs-HOFHN represents resting state high-order functional hypernetwork; star expansion represents resting state functional hypernetwork based on star expansion method; LASSO represents resting state functional hypernetwork constructed by LASSO method; Elastic net represents resting state functional hypernetwork constructed by Elastic net method; Group LASSO represents resting state functional hypernetwork constructed by group LASSO method. Fusion feature represents local properties combined with subgraph feature. BAC represents balanced accuracy.*

## Discussion

Network construction is critical for the classification of brain networks based on hypergraphs. Hypernetwork construction methods have been proposed previous publications; however, most of the existing functional hypernetwork models were characterized by the interaction of multi-regions in a static form (Jie et al., 2016; Li et al., 2017; Zu et al., 2018; Li Y. et al., 2019). However, research has shown that even in the resting state, brain neural activity still reveals transient and subtle dynamic changes. Understanding these dynamic changes is vital if we are to understand the basic characteristics of the brain functional network; these changes may also be significantly correlated with pathological mechanisms in brain diseases (Kudela et al., 2017; Zhao et al., 2020). Therefore, considering this problem, we extended the static brain function hypernetwork model and proposed the construction of a high-order resting state brain function hypernetwork. Here, the window method was first used to reflect the dynamic changes of functional connections in the resting state. Then, the sparse group LASSO method was introduced to construct a high-order brain function hyper network. Using this strategy, we can simultaneously reflect the temporal dynamics of the human brain’s working mechanism and the multivariate interactivity of space. In addition, we also introduced local topological attributes and global information to jointly characterize the high-order brain function hypernetwork, so as to reflect complete topological information relating to the high-order brain function hypernetwork and enhance the ability to detect differences between groups.

For local topological attributes, we used the non-parametric KS test to identify local difference features (corrected by the FDR method), including 21 significantly different functional connections. We also counted the number of occurrences of each independent component in the abnormal functional connection. The results showed that the top four abnormal independent components with the highest frequencies were IC8, IC10, IC18, and IC25. Of these, the corresponding inherent connection networks were the sensory motor network, the visual network, the default mode network and the attention network. For global characteristics, to ensure balance in the number of features between the two groups of subjects, we, respectively, selected the 36 discriminant subgraphs in each group of subjects with the highest frequency difference. Similar to the local features, the number of occurrences of each independent component in the discriminant subgraph were also counted. The results showed that the top four discriminative independent components with the most occurrences were IC8, IC15, IC18, and IC40. Two types of features had fewer overlapping components based on the most discriminative independent components, only IC8 and IC18. This indicated that two types of features complement each other and provide biomarkers related to disease pathology in a more comprehensive manner. Moreover, we found that the discriminative components derived from the two sets of characteristics were located in all of the inherent connection networks, thus indicating that the pathological mechanism of depression relates to damage to the brain’s inherent connection network and is caused by different degrees of abnormalities in different areas of the brain. Previous studies reported similar findings in that patients with depression possessed abnormal connection patterns in different inherent brain connection networks. For example, Lin et al. (Wen et al., 2019) performed static functional connectivity and model recognition analysis to detect the connection mode of whole brain functional networks based on depression and normal people, which showed that there found abnormal intra-network and inter-network connections. Therefore, from the perspective of the inherent connection network, the results of the present research are consistent with previous studies. Next, we discussed the discriminative components from the perspective of brain regions. IC18 and IC8 were found to be most discriminative independent components in the two groups of features. Therefore, we focused on the regions covered by these two components. IC8 included mainly left precentral gyrus. IC18 included mainly left lingual gyrus and left superior frontal gyrus, medial. These differential regions have been confirmed by existing studies to be significantly associated with pathological studies of depressive, and were imaging biomarkers that could not be ignored in the diagnosis of depression. Jin et al. (2011) used graph theory to assess the topological features of brain functional networks in depressed adolescents. They found that brain regions such as the left medial superior frontal gyrus were severely disrupted in depressed adolescents. Lord et al. (2012) studied changes in the community structure of resting-state functional connectivity in unipolar depression. They found changes in brain regions such as the left lingual gyrus. Geng et al. (2019) investigated the neural basis of MDD with somatic symptoms based on the measure of regional homogeneity (ReHo). The result showed that the somatic depression exhibited lower ReHo in the right middle frontal gyrus and left precentral gyrus. Therefore, the discriminative brain regions obtained by the present research were consistent with previous studies.

We applied a high-order functional hypernetwork, a high-order functional network, a functional hypernetwork and a traditional simple binary network models to 38 patients with MDD and 28 NC subjects for classification. The results showed that the high-order brain functional hypernetwork achieved the highest classification accuracy (Table 3). The underlying reason for this is that this particular network model not only considered the complex interactions among multiple components but also consider the dynamic changes of functional connections. The high-order functional network considered the dynamic changes of functional connections, but ignored the complex interactions among multiple components; that is, it only captured pairwise-related information between functional connections. In this way, the constructed network could be too strict, thus leading to the loss of some interactive information between multiple components. Consequently, this network would be unable to accurately characterize the interactions within the human brain (Bullmore and Sporns, 2009; Jie et al., 2014). Conversely, the brain functional hyper-network only considered the interaction between multiple components in the brain but ignored the abundant temporal information contained in the functional connections. This also meant that the constructed network was unable to provide more information relating to brain organization (Leonardi et al., 2013). This result showed that the functionally complex interactions of the brain would not be effectively simulated when considering the interactions between multiple brain regions from space or the time-varying nature of neural interactions from time. Only by considering the multiple interaction effects of the brain in space and the time-varying effects in time, can the complex interaction information of the brain be accurately simulated. In addition, we also compared the classification performance of the high-order hypernetwork model with the hypernetwork models in previous researches. The results show that the rs-HOFHN model proposed in this paper achieves the best classification performance. This also verified the conclusion we got above. That is, considering only the interactions of multiple components of the brain without considering the abundant temporal information contained in the functional connections, it could not more accurately simulate the complex interactions of the brain.

**FIGURE 7:**
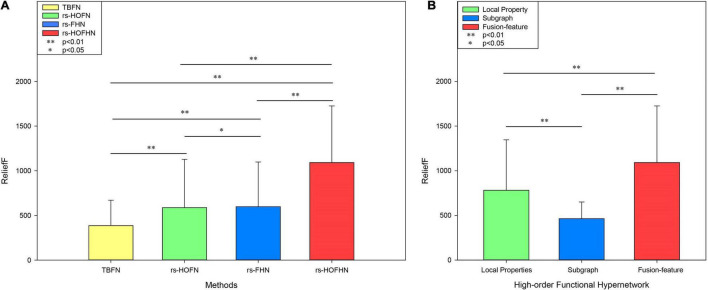
ReliefF weights in different networks and different types of features. **(A)** ReliefF weights for different networks. The *Y*-axis represents the ReliefF weight and the X-axis indicates different networks. TBFN denotes the ReliefF weight of the corresponding local properties and subgraph features obtained from the traditional simple binary functional network. rs-HOFN denotes the ReliefF weight of the corresponding local properties and subgraph features obtained from the high-order functional network. rs-FHN denotes the ReliefF weight of the corresponding local brain regions and the subgraph features obtained from the functional hypernetwork. rs-HOFHN denotes the ReliefF weight of the corresponding local brain regions and subgraph features obtained from the high-order functional hypernetwork. **(B)** The ReliefF weight acquired by different feature extraction methods in the high-order functional hypernetwork. Local property indicates the ReliefF weight obtained by local property features. Subgraph indicates the ReliefF weight obtained by subgraph features. Fusion-feature indicates the ReliefF weight obtained by local property features and subgraph features. ^**^ Represents the *P*-values obtained by non-parametric permutation tests that were <0.05, while * represents *P*-values obtained by the non-parametric permutation test being <0.01.

Finally, the importance of the features was evaluated by the ReliefF algorithm. This is a feature-weighing algorithm that assigns different weights according to the correlation between each feature and category. The greater the weight of the feature, the stronger the classification ability of the feature and *vice versa* (Kononenko, 1996). In this study, the ReliefF algorithm was used to calculate the feature classification weights obtained in different network models (Figure 7A). The results showed that the feature weight value calculated by the high-order function hypernetwork was higher than that calculated by the function hypernetwork and the high-order function network. This result also implied that without or simply consideration of the time-varying or pluralistic nature of the brain’s working mechanism cannot simulate the multi-level and complex interactions of the human brain under different time-space scales. Only by considering interaction information from multiple brain regions and the time-varying characteristics of the human brain can it accurately simulate the complex working mechanism of the human brain and accurately identify biological markers for psychiatric diseases. Furthermore, the rs-HOFHN model was taken as an example to verify the validity of the fusion feature, where the classification weights were evaluated for local features, subgraph features, and fusion features. We found that the ReliefF weights of the fusion features were significantly higher than the ReliefF weights of the local features and subgraph features (Figure 7B). The potential reason for this is that the fusion features effectively integrated local and global topological information; in other words, while reflecting the information of a single component, the features also represented global topology information in the network model. This result suggested that the simultaneous use of local and global topological information can completely characterize topology information from the high-order hyper-network, so as to achieve a better classification and identify more effective biomarkers.

## The Influence of Parameters

Many parameters were considered in this study. We found that the final classification performance was different when the parameter selection was different. These parameters mainly referred to sliding time window size *l*, sliding time window step size *s*, cluster *k*, function hypernetwork construction model regularization parameters (λ1, λ2) and the combination parameter μ in the multi-core learning method. In the next section, we discuss each of these parameters individually.

### Sliding Time Window Size *l*

**FIGURE 8:**
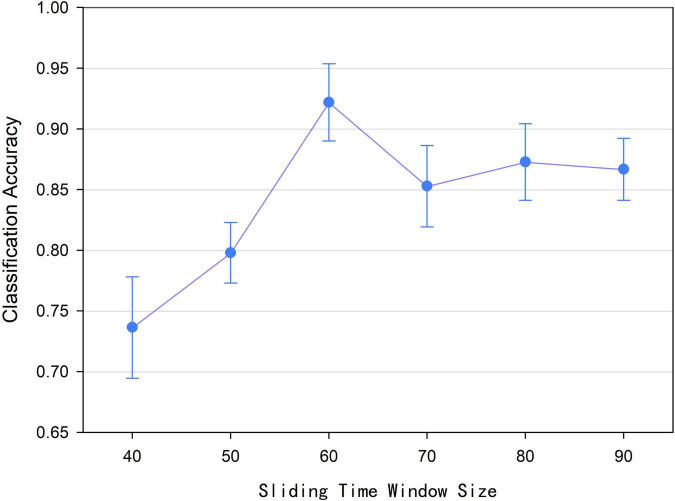
Classification accuracy of different sliding time window sizes.

According to Formula (1), we found that the size *l* of the sliding time window affected the number of time window and the construction of low-order functional networks, which caused the number of time points in the relevant time series and the value of the functional connectivity at a certain moment were different. Accordingly, the construction of the high-order functional hypernetwork was affected ultimately. Therefore, we discussed the effect of sliding time window size on the final classification performance. In this experiment, the sliding time window size *l* was set to 40, 50, 60, 70, 80, and 90, respectively. Based on the size of each sliding time window and other parameters being fixed, a high-order brain function hypernetwork was constructed. Then two types of features were extracted and selected. Finally, multi-kernel learning was adopted and the SVM classification was performed. Figure 8 shows that when the sliding time window is 60, the classification performance is the highest. Under the other size windows, the results are lower than the classification results with a sliding time window size of 60. The underlying reason for this is that when *l* was small, similar time series might be divided into different windows, which would lead to too many features were selected. As a result, more redundant features were included, resulting in lower classification results. On the contrary, when *l* was large, the time window would be correspondingly reduced, which would result in insignificant time-varying characteristics. As a result, the reliability of the network model was affected, leading to lower the classification accuracy.

### Sliding Time Window Step Size *s*

**FIGURE 9:**
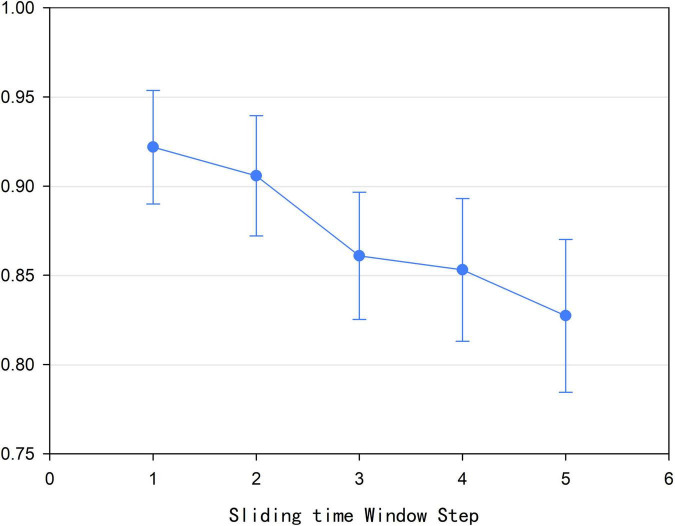
Classification accuracy of different moving steps.

According to Formula (1), we found that size *l* of the sliding time window affected the construction of the high-order brain function hypernetwork. In addition, we also found that the step size *s* of the sliding time window could also influence the construction of the high-order function hyper network model. In this experiment, the time window step size was set to 1, 2, 3, 4, and 5, respectively. Based on each step size for the sliding time window and other parameters being fixed, a high-order brain function hypernetwork was constructed. Then, two types of features were extracted and selected. Finally, multi-kernel learning was adopted and SVM classification was performed. Figure 9 shows that when the step size was 1, that is, the current time window and the next time window were separated by one time point, the classification result was the highest. Moreover, as *s* became larger, the classification result became lower. The underlying reason for this is that as *s* became larger, the number of time windows divided decreased; this meant that the time-varying characteristics of the high-order brain function hypernetwork were not fully reflected. As a result, the reliability of the high-order brain function hypernetwork model was affected, thus leading to a lower classification accuracy.

### The Number of Clusters *k*

**FIGURE 10:**
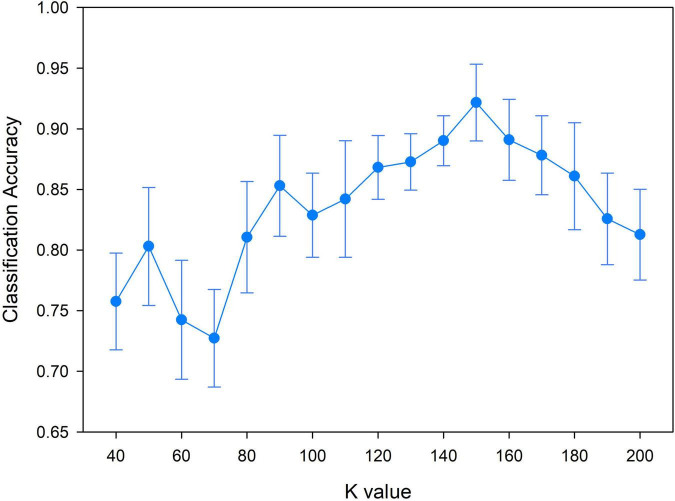
Classification accuracy of different *k* values based on the high-order functional hypernetwork.

The sparse group LASSO method is a preset group selection method. Different groups (different clusters of *k*) would affect the construction of the resting brain function hypernetwork, thus resulting in a different classification performance. Therefore, we changed the cluster number *k* value from 40 to 200 with a step size of 10 to select the appropriate cluster *k* and then constructed the high-order functional hypernetwork. Specifically, for each *k* value, we fixed other parameters and constructed the resting state high-order function hypernetwork based on the sparse group LASSO method. Then, two types of features were extracted and selected. Finally, multi-kernel learning was adopted and SVM classification was performed. In addition, because the random selection of the first initial seed point would cause differences in the network topology, it was necessary to eliminate the influence of initial seed points in the clustering algorithm. Thus, under the condition of each cluster *k* value, 50 experiments were carried out. Then, we selected the average value of the 50 experiments as the final classification result. Figure 10 indicates that the classification accuracy was the highest when *k* = 150 (92.18%).

### Regularization Parameters λ_1_ and λ_2_

Previous studies have shown that parameter λ affects the topology of the network. The regularization parameter λ is known to determine the sparsity and scale of the network. If the λ value is too small, then the created network model would be too rough and involved too much noise; if the λ value was too large, then the network model would be sparser (Lv et al., 2015). Different parameter λ settings are also known to have a certain impact on the reliability of the network topology, especially modularity (Li and Wang, 2015). In addition, λ can affect classification performance and is known to be very sensitive. In other words, if the regularization parameter λ was different, the classification performance would be significantly different (Qiao et al., 2016). Therefore, obtaining the optimal regularization parameter λ value is indispensable for the creation of the network model and the improvement of classification performance. Over recent years, researchers have tried to optimize the reliability of network topology and classification performance by selecting parameter λ (Braun et al., 2012; Li and Wang, 2015; Qiao et al., 2016). However, recent studies have confirmed that it is difficult to obtain high reliability values for network topology when selecting a single λ. Only when the parameter λ value was set to 0.01 can network topology achieve high reliability (very close to 0, meaning that almost all nodes were connected at a hyperedge and that the network was a fully connected network) (Li and Wang, 2015).

**FIGURE 11:**
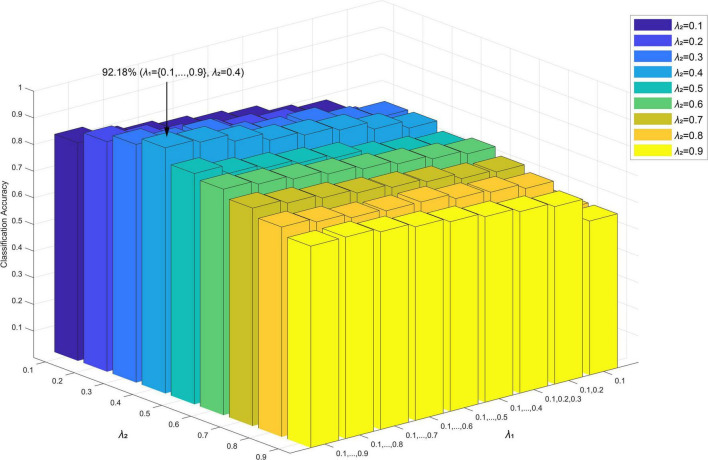
Classification accuracy of different network construction parameters (λ_1_,λ_2_) in the sparse group LASSO method.

Considering this problem, a multi-level regularization parameter setting method was proposed (Jie et al., 2016). In contrast to the single λ setting, a multi-level regularization parameter setting method could set a suite of regularization parameters, thus avoiding the selection of a single λ setting method and thus providing more network topology information. Therefore, in the present study, we used the multi-level parameter setting method to comprehensively characterize the topology of a high-order functional hypernetwork. Considering the time consumption required by the enumeration method, we set the range of regularization parameters (λ1, λ2) as (0, 1) and adopted a series of ascending combinations to construct a high-order function hypernetwork. In other words, when fixing the regularization parameter λ2, the regularization parameter λ1 was changed within the corresponding range to generate the corresponding hyperedge. Specifically, λ2 was set to 0.1,0.2,…,0.9 separately, and λ1 was applied to a series of ascending combinations, namely {0.1}, {0.1,0.2}, {0.1,0.2,0.3},…, {0.1,0.2,…,0.9}, to create hypernetwork models with different high-order resting states. Then, two types of features were extracted and selected. Finally, multi-kernel learning was adopted and SVM classification was performed. The results are shown in Figure 11; the classification accuracy was highest (92.18%) when λ2 = 0.4 and λ1 was used. It should be noted that when λ1 was set to{0.1}, regardless of the value of λ2, the classification result was no higher than 60%. The underlying reason for this is that if λ1 was a single value, there would be some nodes that exist and only exist in a hyperedge; this would result in the HCC^3^ and HCCPN values being unsolvable, so that the corresponding attribute features would be missing and could not be used to construct the classification model.

### The Effectiveness of Each Introduced Local Property

We conducted ablation experiments from two levels of classification performance and feature classification weight to verify the effectiveness of each local feature introduced in this paper. Specifically, we removed each local attribute feature separately, and connected the remaining local attribute features as final local property feature. Then we used the KS nonparametric test method to perform feature selection, where the feature with *p* < 0.05 was selected as the difference feature for classification. The result is shown in Supplementary Table 3. The results show that if any one of the five local attributes was removed, the classification results were lower than those obtained by the five local attributes. Therefore, from the level of classification performance, all local properties introduced in this study were effective.

In addition, to further illustrate the effectiveness of the local fproperties introduced in this paper, we adopted the ReliefF algorithm to measure the effectiveness of the selected features. We removed each local attribute separately, and calculated the classification weights of the difference features obtained from the remaining topological attributes. Then we compared these classification weights with the classification weights obtained from the five local attribute difference features. The results show that after removing HCC1, the classification weight of the remaining topological attribute was 632.61. Similarly, HCC2 was 591.94; HCC3 was 531.29; SP was 550.43; HCCPN was 722.35 (The classification weights of these five groups of local attributes were all statistically significant). The results illustrated that after any local property feature was removed, the result obtained was lower than the classification weight (781.57) obtained by the difference features of five local attribute. Therefore, from the feature classification weight level, the introduced local properties were effective.

In summary, from the perspective of classification and feature validity, it was concluded that the five topological attributes of HCC1, HCC2, HCC3, SP, and HCCPN contained effective classification information to improve the diagnosis of depression.

### Combination Parameter μ in Multi-Kernel Learning

**FIGURE 12:**
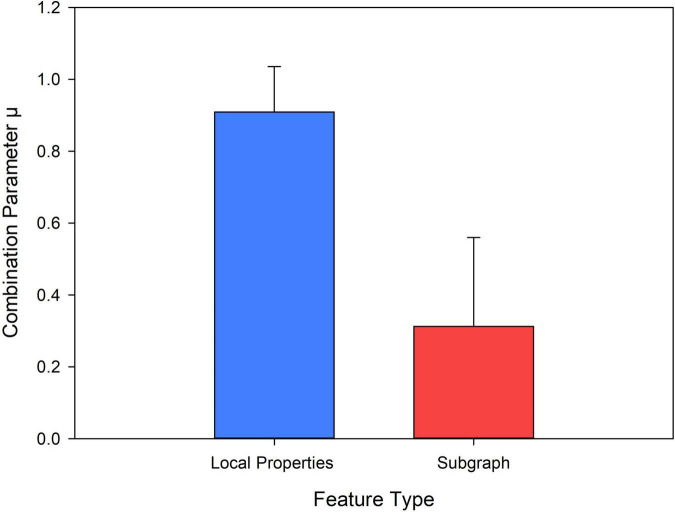
Corresponding combination parameters under different discriminative features.

The most important step in multi-kernel learning is the determination of the combined parameter μ, which directly affects the way that data fusion can influence the classification performance. In the current study, an alignment maximization algorithm was used to determine the weight of the parameterμ_*l*_. Figure 12 shows the average value of the kernel parameters corresponding to two sets of topological attributes during the leave-one-out cross-validation process (Local properties: 0.911; Subgraph: 0.31). Under this value, the classification result of the proposed method reached 92.18%. Note that the sum of the squares of these two weights was not equal to 1, whose underlying reason was that we calculated the average value of the kernel parameters during the leave-one-out cross-validation process. In some cases, the weight of a parameter maybe a negative value, which made the sum of the squares of these two weights not being exactly equal to 1.

### Repetitive Verification

To further verify the effectiveness of the proposed method, we used the Alzheimer’s Disease Neuroimaging Initiative (ADNI) data set^5^ to perform the current study. Normal subjects and patients with Alzheimer’s disease (AD) were selected from the database, including 30 normal subjects and 29 Alzheimer’s patients. A preprocessing process, that was similar to the process used for the MDD data set was utilized; this consisted of time layer correction, head motion correction, spatial normalization, linear dimensionality reduction, band pass filtering and smoothing. Then, the GICA method was used to divide the independent components and extract the spatial distribution and time series of each independent component. In the AD data set, 23 independent components were obtained. Based on the average time series data relating to independent components, a rs-HOFN model was constructed based on the time sliding window method, a rs-FHN model was constructed based on the sparse group LASSO method, and a rs-HOFHN model was constructed based on the time sliding and sparse group LASSO method. Next, local attributes (rs-HOFN: node degree, betweenness centrality and node efficiency; rs-FHN and rs-HOFHN: HCC, HCCPN and SP) and global information (HOFH: frequent subgraph features was extracted using the gSpan algorithm; rs-FHN and rs-HOFHN: hyperedges were regarded as subgraph features) were extracted. Furthermore, the non-parametric KS test and FSFS methods were applied to select local difference features and discriminant subgraphs. Finally, multi-kernel learning was used to fuse features and the SVM classifier was used to perform classification. The classification results are shown in Supplementary Table 4. These results showed that the rs-HOFHN model obtained better classification performance, thus indicating that a resting state high-order functional hypernetwork can more accurately describe the functional connections of the human brain, characterize the complex working mechanisms of the human brain, and identify more accurate pathological markers.

## Conclusion

Previous research showed that a functional hypernetwork could capture interactions among multiple regions in a static form but ignored the dynamic changes of the functional connections over a short period of time. Therefore, considering the time variability of neural activity, we constructed a high-order function hyper network to meet the time variability of human brain interactions in time and the multiple interaction capabilities in space. In addition, local topological attributes and global characteristics were introduced simultaneously to fully characterize the high-order brain function hypernetwork model. Then, the two sets of features were mixed into a mixed kernel through multi-kernel learning for classification and diagnosis.

In this study, we identified the most discriminative functional connections and discriminant subgraphs in MDD. We found that our findings were consistent with those published previously. We evaluated the classification performance of a high-order hyper network, a traditional hypernetwork, a high-order network and a traditional binary network. We found that the high-order hyper network achieved the best classification performance, thus implying that a better classification performance could be achieved if the multivariate interactions and time-varying characteristics of neural interactions were considered simultaneously. In addition, two sets of features and multiple features were evaluated separately. We found that the classification accuracy and relief weighting of multi-features were better than single features (i.e., local attribute features and subgraph features), thus suggesting that better classification could be achieved, and more effective biomarkers could be identified when both local features and global information were used to jointly characterize high-order functional networks.

However, this study has some limitations that need to be considered. The method used to construct the functional hypernetwork in this research involved non-overlapping groups. However, recent study have shown that the overlap between groups may affect the construction of function brain hypernetwork models, thereby affecting the effectiveness of the classification model (Shen et al., 2019). Therefore, the overlap between groups (such as the overlapping group LASSO method) can be considered to further improve the construction of the hypernetwork in future research. In addition, we needed to perform clustering before constructing the high-order functional hypernetwork. Although different k values were selected, and multiple experiments based on a specific k value were performed, it is not possible to eliminate the effect of initial random seed points on the constructed brain function hypernetwork and classification results. Therefore, further research is needed to select a more optimal parameter setting method to generate a more stable hyperedge and further improve the topology of the function hypernetwork. Finally, in our experiment, it is mainly assumed that the hyperedge was decomposable in nature. That is, we decomposed the hyperedge into multiple nodes and tend to associate these nodes because they have common membership in the same hyperedge. In future research, we can try to introduce the line graph (Bandyopadhyay et al., 2020) theory of the hypergraph that regard the hyperedge as a node to consider its hyperedge information, thus performing the classification and diagnose of brain diseases.

## Data Availability Statement

The raw data supporting the conclusion of this article will be made available by the authors, without undue reservation.

## Ethics Statement

The studies involving human participants were reviewed and approved by the medical ethics committee of Shanxi Province (reference number: 2012013). Written informed consent to participate in this study was provided by the participants’ legal guardian/next of kin.

## Author Contributions

YL was responsible for the study design and writing the manuscript. QL, TL, and ZZ performed the statistical analysis. YX and YY provided and integrated the experimental data. HG and JC provided conception and design of the work. All authors approved the final version of the manuscript.

## Conflict of Interest

The authors declare that the research was conducted in the absence of any commercial or financial relationships that could be construed as a potential conflict of interest.

## Publisher’s Note

All claims expressed in this article are solely those of the authors and do not necessarily represent those of their affiliated organizations, or those of the publisher, the editors and the reviewers. Any product that may be evaluated in this article, or claim that may be made by its manufacturer, is not guaranteed or endorsed by the publisher.

## Footnotes

1
http://www.fil.ion.ucl.ac.uk/spm

2
http://mialab.mrn.org/software/gift

3
http://www.cis.hut.fi/projects/ica/icasso

4
http://www.csie.ntu.edu.tw/~cjlin/libsvm/

5
http://adni.loni.usc.edu/
