# Supplementary material for: Construction and Multiple Feature Classification Based on a High-Order Functional Hypernetwork on fMRI Data
Source: Front Neurosci. 2022 Apr 13;16:848363. doi: 10.3389/fnins.2022.848363 (PMC9043754; doi:10.3389/fnins.2022.848363)
Supplement: Supplementary file 1 [file Data_Sheet_1.docx]

**Supplemental Text S1.** **Group independent component analysis.**

Let $Y_{i}$ be the$T\times V$ preprocessed and intensity-normalized functional imaging data matrix for subject i, where T time points over V voxels are collected on *M* subjects. For GICA, the data first undergo reduction and whitening at the subject and group levels using principal component analysis (PCA). Let $Y_{i}^{*}=F_{i}^{-}Y_{i}$ be the $T_{1}\times V$ PCA-reduced data for subject i, where $F_{i}^{-}=Y_{i}^{T}$ is the $T_{1}\times T$ standardized reducing matrix and $T_{1}$ is the number of principal components retained for each subject. Note that $Y_{i}$ has rows of zero mean (we remove the mean across space for each time point) to improve conditioning of the covariance matrix, however this step has no effect on the PCA reduction or GICA decomposition. Let$Y_{i}^{*}=\left[Y_{1}^{*T},\ldots,Y_{M}^{*T}\right]^{T}$be the $MT_{1}\times V$ time-concatenated aggregate data. Group-level PCA is required to reduce the dimension of the data to the number of components to be estimated with ICA. Let the$T_{2}\times V$PCA-reduced aggregate data be $X\equiv G^{-}Y^{*}=\left[G_{1}^{T},\ldots,G_{M}^{T}\right]\left[\left(F_{1}^{-}Y_{1}\right)^{T},\ldots,\left(F_{M}^{-}Y_{M}\right)^{T}\right]^{T}=\sum_{i=1}^{M} G_{i}^{T}F_{i}^{T}Y_{i}=\sum_{i=1}^{M} X_{i}$,where $G^{-}$ is the $T_{2}\times MT_{1}$ standardized reducing matrix. Following square noise-free spatial ICA estimation, we can write $X=\hat{A}\hat{S}$ where the generative linear latent variables$\hat{A}$  and $\hat{S}$are the $T_{2}\times T_{2}$ mixing matrix related to subject time courses (TCs) and the $T_{2}\times V$ aggregate SM, respectively.

To estimate subject-specific spatial maps (SPs) and TCs, we used the recently developed GICA3 back-reconstruction method based on PCA compression and projection. In GICA3, the subject-specific SM is defined as $\tilde{S}_{i}\equiv\hat{A}^{-}G_{i}^{T}F_{i}^{T}Y_{i}$ which yields exactly that the aggregate SM is the sum of the subject-specific SMs, $\hat{S}\equiv\sum_{i=1}^{M} \tilde{S}_{i}$.This is analogous to a random effects model where the subject-specific effects are zero-mean distributed deviations from the group mean effect. The natural estimator of subject-specific TCs is $\hat{R}_{i}\equiv F_{i}\left(G_{i}^{T}\right)^{-}\hat{A}$. The product of each subject-specific TC and SM is a perpendicular projection of the data onto the PCA column space, $\hat{R}_{i}\equiv F_{i}\left(G_{i}^{T}\right)^{-}\hat{A}$. The PCA compressed fitted values are exactly the PCA compressed data ***X***, that is, the fitted values and data agree in the PCA space. The fitted compressed values$\tilde{X}_{i}\equiv\hat{A}\tilde{S}_{i}=X_{i}$, are a product of the subject-specific TC and SM, and similarly for the mean fitted compressed value,$\tilde{X}_{i}\equiv\sum_{i=1}^{M} \tilde{X}_{i}=X$. Thus, it is the amount of information retained in the PCA steps that largely determines the subject-specific TC and SM estimates. Compared with dual regression, GICA3 provides more robust and accurate estimates of subject-specific components.

GICA was performed using the GIFT (http://mialab.mrn.org/software/gift) toolbox. Based on fMRI data, 54 independent components (ICs) were estimated. Next, we illustrated the rationality of selecting 54 ICs. For ICA, extensive feature is the uncertainty of the number of independent components (ICs) [1]. A review was listed including 47 recent studies on ICA in fMRI datasets. Some of these studies were reviewed in [1] and [2]. Additional researches published in the past four years (2016–2020) were included (See Supplemental Table S1). Through summary, it is found that there is no gold standard for determining the numbers of ICs. In different studies, the numbers of ICs used vary widely, up to 150 ICs and at least 8 ICs. Among them, there are 16 articles with ICs greater than 50.

Moreover, through summary, it is found that GIFT toolbox and MELODIC (multivariate exploratory linear decomposition) toolbox were commonly used to determine the number of independent components based on fMRI data in existing studies, of which there were 27(57.45%) studies that selected GIFT toolbox to calculate the number of independent components. The minimum description length criterion (MDL) was adopted in the GIFT. Considering the spatial correlation, the number of best decomposed components in all subjects was automatically estimated. In this process, mainly based on the input of pre-processed image data, directly through a specific algorithm to pre-determine the number of the best components, rather than artificial settings. Further, it was considered appropriate that when optimal number of ICs is approximate one-fourth to one-fifth of the number of the time points [3]. In our experiment, after removing the first 10 time points, there were 238 remaining time points, so the appropriate range of independent components was 47-59, 54 components (number of ICs identified in our study) being located in it. Because the independent components extracted using group independent component analysis included not only brain network components of interest in this paper, but also other unrelated components or components with more noise. Because of this, the study was further screened and confirmed by prior template matching method and manual inspection method. Finally, 32 unrelated components were removed and 22 components were retained to participate in the post-study.

**Reference**

[1] Wang, Y. and T.Q. Li, Dimensionality of ICA in resting-state fMRI investigated by feature optimized classification of independent components with SVM[J]*.* Frontiers in Human Neuroscience. 9.

[2] Dennis, E.L. and P.M. Thompson, Functional Brain Connectivity Using fMRI in Aging and Alzheimer’s Disease[J]*.* Neuropsychology Review. 24(1): p. 49-62.

[3] Greicius, M.D., G. Srivastava, A.L. Reiss, et al., Default-mode network activity distinguishes Alzheimer's disease from healthy aging: evidence from functional MRI[J]*.* Proceedings of the National Academy of Sciences, 2004. 101(13): p. 4637-4642.
