# Supplementary material for: Construction and Multiple Feature Classification Based on a High-Order Functional Hypernetwork on fMRI Data
Source: Front Neurosci. 2022 Apr 13;16:848363. doi: 10.3389/fnins.2022.848363 (PMC9043754; doi:10.3389/fnins.2022.848363)
Supplement: Supplementary file 2 [file Data_Sheet_2.docx]

**Supplemental Text S2. Reasons to use the sparse group LASSO method to build hypernetworks**

The existing method used to construct a functional hypernetwork predominantly includes two methods that are either based on star expansion [1, 2] or the sparse representation method [3, 4]. However, the star expansion method is associated with difficulties when defining the effective number of nearest neighbors or the search radius and may be sensitive to noise, thus restricting the performance of data modeling [5]. In contrast to the star expansion method, the sparse representation method is used to represent the centroid vertex by the linear combination of its neighbors; then, only the vertices with non-zero coefficients to the centroid vertex are used to generate the hyperedge. This method can overcome the limitations of star expansion and permits data adaptation and noise robustness [6]. Therefore, this method has been widely recognized by researchers and applied in brain disease research.

Problems associated with the sparse representation method can be solved in a variety of different ways. Different penalties have different methods for solving sparse representation models and a variety of methods have been proposed to solve the sparse representation model to allow construction of a brain function hypernetwork, such as the LASSO method [3], the elastic net method [7], the group LASSO method [8], and the sparse group LASSO method [9]. Research has shown that that the sparse group LASSO method can achieve the best classification performance [9] and can construct a functional brain hypernetwork in the most efficient manner. Therefore, we used the sparse group LASSO method to create a high-order functional hypernetwork for the resting brain.

**References**

[1] Li Y, Gao X, Jie B, Yap P-T, Kim M, and Wee C-Y, *Multimodal hyper-connectivity networks for mci classification*, in (International Conference on Medical Image Computing and Computer Assisted Intervention. 2017.

[2] Zu C, Gao Y, Munsell B, Kim M, Peng Z, Cohen JR, et al. Identifying disease-related subnetwork connectome biomarkers by sparse hypergraph learning[J]. Brain Imaging and Behavior, 2018. 13(4): 879-892.

[3] Jie B, Wee CY, Shen D, and Zhang D. Hyper-connectivity of functional networks for brain disease diagnosis[J]. Medical Image Analysis, 2016. 32: 84.

[4] Li Y, Liu J, Gao X, Jie B, and Shen D. Multimodal hyper-connectivity of functional networks using functionally-weighted lasso for mci classification[J]. Medical Image Analysis, 2019. 52: 80-96.

[5] Zhang Z, Lin H, and Gao Y. Dynamic hypergraph structure learning [C]//. international joint conference on artificial intelligence. 2018.

[6] Liu Q, Sun Y, Wang C, Liu T, and Tao D. Elastic net hypergraph learning for image clustering and semi-supervised classification[J]. IEEE Transactions on Image Processing, 2017. 26(1): 452-463.

[7] Liu Q, Sun Y, Wang C, Liu T, and Tao DJIToIPAPotISPS. Elastic net hypergraph learning for image clustering and semi-supervised classification[J]. 2016. 26(1): 452-463.

[8] Guo H, Li Y, Xu Y, Jin Y, Xiang J, and Chen J. Resting-state brain functional hyper-network construction based on elastic net and group lasso methods[J]. Frontiers in neuroinformatics, 2018. 12: 25-25.

[9] Li Y, Sun C, Li P, Zhao Y, and Chen J. Hypernetwork construction and feature fusion analysis based on sparse group lasso method on functional fmri dataset[J]. Frontiers in Neuroence, 2020. 14: 60.
