# Supplementary material for: Construction and Multiple Feature Classification Based on a High-Order Functional Hypernetwork on fMRI Data
Source: Front Neurosci. 2022 Apr 13;16:848363. doi: 10.3389/fnins.2022.848363 (PMC9043754; doi:10.3389/fnins.2022.848363)
Supplement: Supplementary file 3 [file Data_Sheet_3.docx]

**Supplemental Text S3. Results of multiple linear regression analysis between network properties and confounding variables.**

| Confounding Variables | Coefficients | Std. Error | T Stat. | P-value | Lower 95% | Upper 95% |
| --- | --- | --- | --- | --- | --- | --- |
| Clustering Coefficient HCC^1^ (Adj. R_sqr_ = -0.018_,_ P = 0.613) | | | | | | |
| Intercept | 1.1890 | 0.1077 | 11.0359 | <0.0010 | 0.9737 | 1.4044 |
| Gender | 0.0094 | 0.0418 | 0.2240 | 0.8235 | -0.0742 | 0.0929 |
| Age | 0.0005 | 0.0021 | 0.2254 | 0.8224 | -0.0037 | 0.0047 |
| Educational Attainments | 0.0187 | 0.0147 | 1.2723 | 0.2080 | -0.0107 | 0.0482 |
| Clustering Coefficient HCC^2^ (Adj. R_sqr_ = 0.002_,_ P = 0.423) | | | | | | |
| Intercept | 1.5293 | 0.1220 | 12.5356 | <0.0010 | 1.2854 | 1.7731 |
| Gender | -0.0664 | 0.0473 | -1.4042 | 0.1653 | -0.1610 | 0.0281 |
| Age | 0.0020 | 0.0024 | 0.8342 | 0.4074 | -0.0028 | 0.0068 |
| Educational Attainments | 0.0167 | 0.0167 | 0.9991 | 0.3216 | -0.0167 | 0.0500 |
| Clustering Coefficient HCC^3^ (Adj. R_sqr_ = -0.041_,_ P = 0.942) | | | | | | |
| Intercept | 1.0435 | 0.1389 | 7.5105 | <0.0010 | 0.7658 | 1.3212 |
| Gender | 0.0230 | 0.0539 | 0.4260 | 0.6716 | -0.0848 | 0.1307 |
| Age | -0.0014 | 0.0027 | -0.5198 | 0.6050 | -0.0068 | 0.0040 |
| Educational Attainments | -0.0035 | 0.0190 | -0.1838 | 0.8548 | -0.0414 | 0.0345 |
| Clustering Coefficient HCCPN (Adj. R_sqr_ = 0.032_,_ P = 0.800) | | | | | | |
| Intercept | 0.0097 | 0.0038 | 2.5619 | 0.0129 | 0.0021 | 0.0172 |
| Gender | 0.0010 | 0.0015 | 0.6764 | 0.5013 | -0.0019 | 0.0039 |
| Age | 0.0000 | 0.0001 | -0.4449 | 0.6579 | -0.0002 | 0.0001 |
| Educational Attainments | -0.0004 | 0.0005 | -0.7973 | 0.4283 | -0.0014 | 0.0006 |
| The Shortest Path SP (Adj. R_sqr_ = -0.018_,_ P = 0.610) | | | | | | |
| Intercept | 2.6310 | 0.1905 | 13.8145 | <0.0010 | 2.2503 | 3.0117 |
| Gender | 0.0642 | 0.0739 | 0.8684 | 0.3885 | -0.0835 | 0.2118 |
| Age | -0.0019 | 0.0037 | -0.5023 | 0.6172 | -0.0093 | 0.0056 |
| Educational Attainments | 0.0202 | 0.0260 | 0.7756 | 0.4409 | -0.0318 | 0.0722 |

The range of age is 17–51 years. Optional values of gender are male and female. Optional values of educational attainments are illiteracy, primary school, junior high school, senior high school, junior college, college, graduate degree and above. Adj. R_sqr_, adjusted R square.Coefficients, regression coefficient. Std. Error, standard error. T stat., T statistic. Lower 95%, low bound of 95% confidence limits. Upper 95%, upper bound of 95% confidence limits.
