# Supplementary material for: Construction and Multiple Feature Classification Based on a High-Order Functional Hypernetwork on fMRI Data
Source: Front Neurosci. 2022 Apr 13;16:848363. doi: 10.3389/fnins.2022.848363 (PMC9043754; doi:10.3389/fnins.2022.848363)
Supplement: Supplementary file 4 [file Data_Sheet_4.docx]

# Supplemental Text S4：Weisfeiler-Lehman Subtree Kernel

Given two graphs *G* and *H*, let V_0_ be the original set of node labels of *G* and *H*, and V_𝑖_ be the set of letters that occur as node labels at least once in *G* or *H* at the end of the *i*-*th* (*i*=0,...,*h*) iteration of the Weisfeiler-Lehman algorithm. Assume that all $V_{i}=\{\sigma_{i1},\sigma_{i2},...,\sigma_{i|V_{i}|}\}$ are pairwise disjointed. The Weisfeiler-Lehman subtree kernel on two graphs *G* and *H* with ℎ iterations is defined as follows:

where ; $C{}_{i}(G,\sigma_{ij})$represents the number of occurrences of the node label $\sigma_{ij}$of graph *G* in the *i*-th iteration.; $C{}_{i}(H,\sigma_{ij})$ represents the number of occurrences of the node label $\sigma_{ij}$of graph *H* in the *i*-th iteration. Note that the graph in current study was the undirected graph.

Given two graphs, the specific process for the Weisfeiler-Lehman test of graph isomorphism is: (1) if these two graphs were unlabeled graphs, that is, nodes of the graphs were not allocated labels, the specific node was labeled via the number of edges that are linked with the node. (2) the label of each node was updated according to its last label and the labels of its neighbors. In other words, the updated node labels of each node were compressed into a novel and shorter label. (3) iterated step (2), until the node label sets were same, or the number of iteration reaches its predefined maximum value. The specific algorithm was as follows, taking one iteration of the 1-dim as an example.

**Algorithm 1**

One iteration of the 1-dim Weisfeiler-Lehman test of graph isomorphism.

| 1 Step 1: Multiset-label determination  2 For *i*=0, set $M_{i}\left( v \right)=l_{0}\left( v \right)=l(v)$ 3 For *i*>0, allocate a multiset-label $M_{i}(v)$ to each node *v* in G and G’ which compried of the  multiset $\{l_{i-1}\left( u \right)\vert u\in N\left( v \right)\}$ 4 Step 2: Sorting each multiset  5 Sort values in $M_{i}\left( v \right)$ in ascending order and link them into a string $s_{i}\left( v \right)$ 6 Add $l_{i-1}\left( v \right)$ as a prefix to $s_{i}\left( v \right)$ and call the resulting string $s_{i}\left( v \right)$ 7 Step 3: Label compression  8 Sort all of the strings $s_{i}\left( v \right)$ for all *v* from G and G’ in ascending order  9 Use a function $f:\Sigma^{*}=\Sigma$ map each string $s_{i}\left( v \right)$ to a new compressed label.  10 Step 4: Relabeling  11 Set $l_{i}\left( v \right):=f(s_{i}(v))$ for all nodes in G and G’ |
| --- |

A specific example was showed in Figure 1.


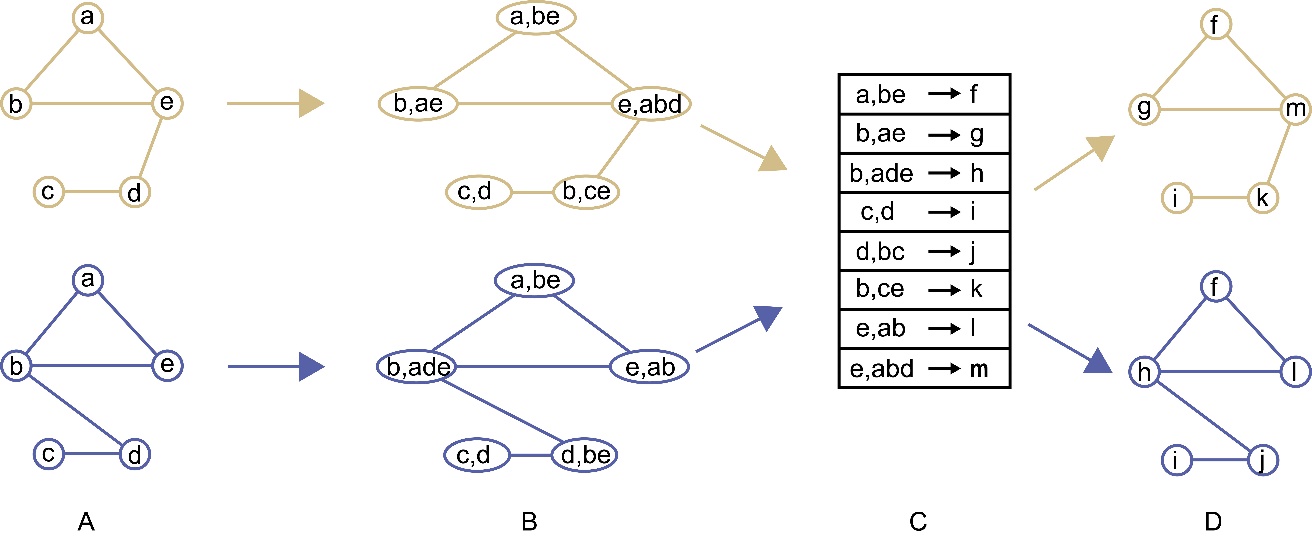


Figure 1 Example of the construction process for the Weisfeiler-Lehman subtree kernel. *G* and *H* represent the given two graphs. The label set is *L*= {*a*, *b*, *c*, *d*, *e*, *f*, *g*, *h, i*, *j*, *k*, *l, m*}. (A) the initial labeled graphs, (B) augmented labels, (C) label compression, (D) relabled networks.

Figure 1 shows the construction process for the Weisfeiler-Lehman subtree kernel when the iteration time is one time. The label set is *L*= {*a*, *b*, *c*, *d*, *e*, *f*, *g*, *h, i*, *j*, *k*, *l, m*}. = {1,1,1,1,1,1,1,0,1,0,1,0,1} and = {1,1,1,1,1,1,0,1,0,1,0,1,1}..

**References**

1. Shervashidze, N., et al., Weisfeiler-Lehman Graph Kernels. Journal of Machine Learning Research, 2011. **12**(3): p. 2539-2561.
