# Supplementary material for: Construction and Multiple Feature Classification Based on a High-Order Functional Hypernetwork on fMRI Data
Source: Front Neurosci. 2022 Apr 13;16:848363. doi: 10.3389/fnins.2022.848363 (PMC9043754; doi:10.3389/fnins.2022.848363)
Supplement: Supplementary file 5 [file Data_Sheet_5.docx]

# Supplemental Text S5: The selection of the optimal feature number on discriminant subgraph

In this paper, hyperedges were extracted as subgraph features, and the FSFS method was used to calculate the frequency difference and sort them to select the subgraphs corresponding to the top-*k* frequency difference as the discriminant subgraphs for classification. Here, the selection of the *k* value will affect the classification, that is, the number of discriminative subgraph features was different, and the classification was different. Therefore, in this experiment, the number of features was set to 6–50 with a step size of 2. The classification model was respectively constructed and the influence of the number of features on the classification result was explored. The higher the classification accuracy, the stronger the discriminativeness of the group of discriminant subgraphs. As illustrated in Figure 1, the experimental results show that as the number of features increased, the classification accuracy generally tended to decrease after the initial increase. Classification performance was best when the number of features was 36. The potential reason is that on the one hand, when the feature number is too small, the difference between the depression group and the normal group is not well reflected; on the other hand, when the number of features is too large, the redundant features seriously affect the efficiency of the classifier. Thus, we selected the 36 subgraphs as discriminative subgraphs to perform multi-feature fusion.


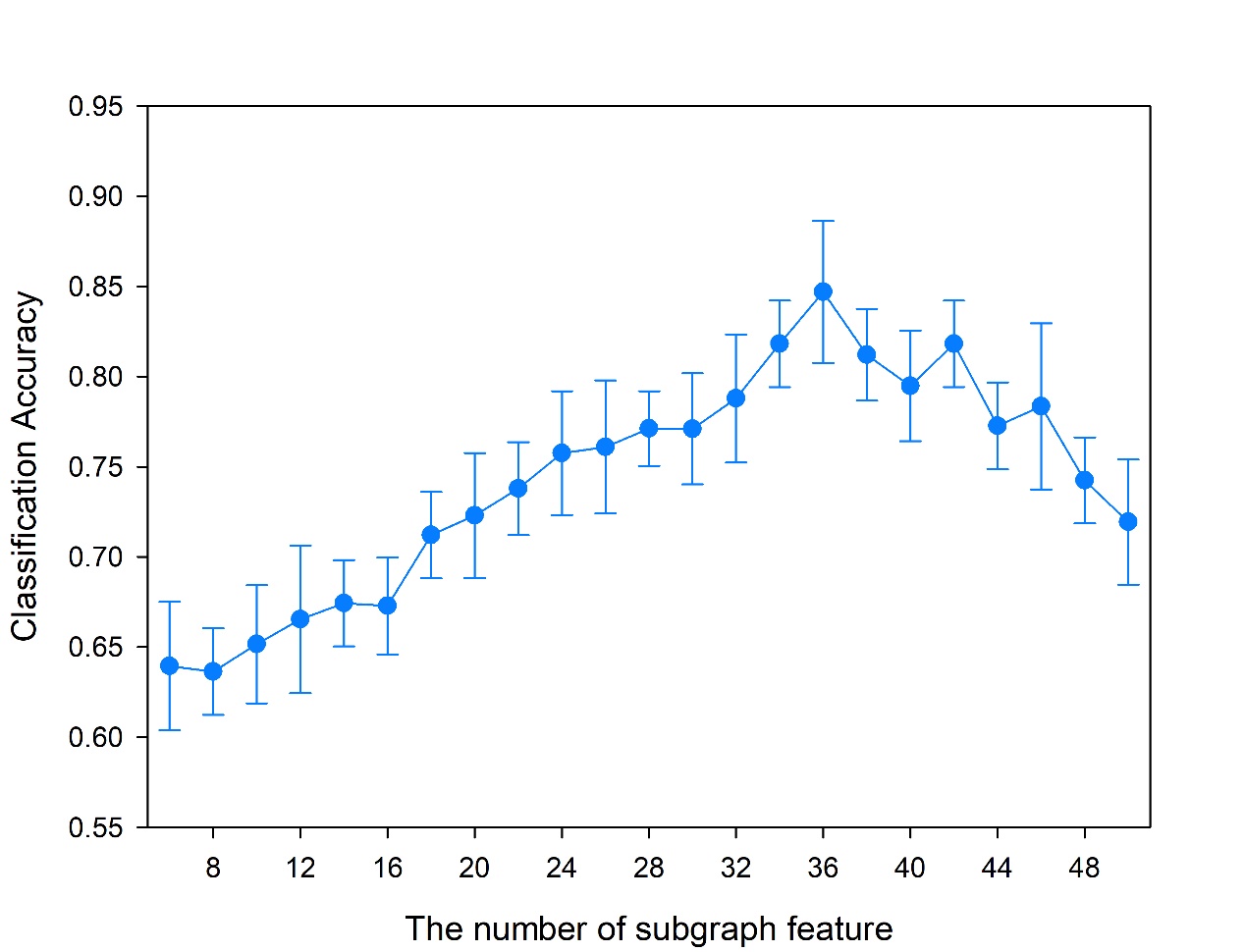


**Figure 1.** **Effect of subgraph feature number on classification performance.** The ordinate indicates the classification accuracy, while the abscissa denotes different feature numbers, ranging from 6–36 with a step size of 2.
