# Supplementary material for: Construction and Multiple Feature Classification Based on a High-Order Functional Hypernetwork on fMRI Data
Source: Front Neurosci. 2022 Apr 13;16:848363. doi: 10.3389/fnins.2022.848363 (PMC9043754; doi:10.3389/fnins.2022.848363)
Supplement: Supplementary file 6 [file Table_1.docx]

**Supplemental Table S1. Comparison among Similar ICA Researches**

| **Paper[year]** | **Image Type** | **Disease/Groups** | **Patients/N** | **Patients/Age** | **Control/N** | **Control/Age** | **ICA numbers** | **Tool** | **Features** |
| --- | --- | --- | --- | --- | --- | --- | --- | --- | --- |
| Greicius, M.D.,  et al [1] 2004 | fMRI | AD | 13 | 68.0 – 83.0 | 28 (14,14) | 66.0 – 89.0  18.0 – 24.0 | 31 | Multivariate Exploratory Linear  Decomposition (MELODIC) | Two-sample t test |
| Sorg, C., et al  [2] 2007 | fMRI | MCI | 24 | 69.3 ± 8.1 | 16 | 68.1± 3.8 | 15,39,54 | GIFT toolbox | Functional brain  networks from ICA |
| Greicius, M.D.,  et al.[3] 2007 | fMRI | MDD | 11 | / | 17 | / | 25 | MELODIC | one-sample t-tests |
| Calhoun, V.D ,et al [4] 2008 | fMRI | Schizophrenia (SP) | 20 | 39.7 ± 10.1 | 20 | 31.2 ± 10.9 | 19 | GIFT toolbox | Functional brain  networks from ICA |
| Sui, J., et al.  [5] 2009 | fMRI | SP | 25 | 32.0 ± 12.0 | 28 | 32.0 ± 13.0 | 8-16 | Joint independent component analysis (jICA) | Spatial features, templates |
| Filippini, N.,  et al.[6] 2009 | fMRI | APOE - ε4 allele | 18 | 28.4 ± 4.9 | 18 | 28.6 ± 3.9 | 25 | MELODIC | Encoding memory paradigm using blood oxygen  level-dependent fMRI |
| Vince, D.,  et al.,[7] 2009 | fMRI | SP | SZ 21  BP 14 | SZ 34.9 ± 11.8  BP33.2 ± 9.9 | 26 | 30.3 ± 8.8 | 30 | GIFT toolbox | one-sample t-test |
| Veer, I.M.,  et al.[8] 2010 | fMRI | MDD | 19 | 36.1 ± 10.6 | 19 | 36.2 ± 9.7 | 20 | MELODIC | t-test, Functional brain  networks from ICA |
| Fan, Y., et al  [9] 2011 | fMRI | SP | 31 | 24.0 ± 6.0 | 31 | 26.0 ± 4.0 | 19 | GIFT toolbox | Functional brain  networks from ICA |
| Ahmed, A.E.,  et al [10] 2011 | fMRI | seasonal affective disorder (SAD) | 45 | 39.8 ± 10.6 | 45 | / | 20-150 | MELODIC | dual regression |
| Petrella, J.R.,  et al [11]2011 | fMRI | MCI，AD | AD12  MCIc11  MCInc20 | 72.0 ± 5.9,  76.2 ± 6.0,  72.3 ± 9.3 | 25 | 70.8 ± 4.3 | 20 | GIFT toolbox | Goodness-of-fit (GOF) indices  of DMN expression |
| Binnewijzend, M.A., et al.[12]  2012 | fMRI | AD | 39 | 67.0 ± 8.0 | 43 | 69.0 ± 7.0 | 27 | MELODIC | Functional brain  networks from ICA |
| Du, W., et al  [13] 2012 | Task fMRI | SP | 28 | 39.4 ± 12.7 | 28 | 31.5 ± 11.7 | 40 | GIFT toolbox | Spatial components  from ICA |
| Damoiseaux, J.S., et al [14] 2012 | fMRI | AD | 21 | 64.2 ± 8.7 | 18 | 62.7 ± 10.3 | 25 | MELODIC | dual regression |
| Fan, T., et al [15]2012 | fMRI | Bipolar depression | 21 | 31.1 ± 8.5 | 21 | 31.6 ± 8.9 | 17 | GIFT toolbox | one-sample  t-tests and two-sample t-tests |
| Arbabshirani, M.R., et al.  [16] 2013 | fMRI | SP | 28 | 39.7 ± 10.1 | 28 | 36.5 ± 11.3 | 20 | GIFT toolbox |  |
| Vanessa, S., et al. [17] 2014 | fMRI | SP | 24 | mean age 35.13 | 29 | mean age 29.5 years | 19 and 25 respectively | MELODIC | 246 features, spatial and  temporal |
| Hoekzema, E.,  et al.[18] 2014 | fMRI | ADHD | 22 | 32.8±10.8 | 23 | 29.3 ± 8.9 | 20 | GIFT toolbox | t-test |
| Maneshi, M.,  et al [19] 2014 | fMRI | Mesial temporal lobe epilepsy (MTLE) | 10 | 29.0 ± 11.0 | 10 | 32.0 ± 9.0 | 30-50 | MELODIC | The func-tional connectivity of each reliable specific resting-state network |
| Kaufmann, T.,  et al.[20] 2015 | fMRI | SP | 71 | 28.2 ± 7.8 | 196 | 31.5 ± 7.8 | 20-80 | MELODIC | Functional brain  networks from ICA |
| Baggio, H.C.,  et al.[21] 2015 | fMRI | Parkinson | 65 (43,22) | 64.0 ± 9.8,  66.1 ± 12.2 | 38 | 63.4 ± 10.5 | 25 | MELODIC | Functional brain  networks from ICA |
| Tessitore, A., et al [22] 2016 | fMRI | Parkinson | 40 (20,20) | 62.1, 61.5, 61.5, 61.2 | 20 | 60.3 -62.1 | 40 | FastICA and the self-organizing group ICA (sogICA)  algorithms | Functional brain  networks from ICA |
| He, H., et al.  [23] 2016 | fMRI | MDD | 53 (13,40) | 35.2 ± 10.3,  35.2 ± 9.3 | 33 | 33.7 ± 10.2 | 75 | GIFT toolbox | one-sample t-test |
| Yang, W.,  et al.[24] 2017 | fMRI | AD | 70 | 77.2 ± 6.2 | 140 | 75.7 ± 3.8 | 27 | GIFT toolbox | t-test, Functional brain  networks from ICA |
| Qureshi, M.N.I., et al.[25] 2017 | fMRI | SP | 72 | 38.2 ± 13.9 | 72 | 38.9 ± 11.7 | 30 | MELODIC | Functional brain  networks from ICA |
| Skåtun, K.C., et al.[26] 2017 | fMRI | SP | 182 | 28.7-51.4 | 348 | 31.9 - 64.4 | 80 | meta-ICA approach - | Functional brain  networks from ICA |
| Wu, X.J., et al.,  [27] 2017 | fMRI | Schizophrenia,MDD | 24 schizo-  phrenia,  20 MDD | schizophrenia  25.3 ±6.2,  MDD  26.6 ±7.7 | 43 | 28.2 ± 7.2 | 30 | GIFT toolbox | Functional brain  networks from ICA |
| Komal, B., et al.,  [28] 2017 | fMRI | Progressive supranuclear palsy (PSP) and corticobasal syndrome  (CBS) | 31 (20,11) | 69.3 ± 4.8,  66.0 ± 3.9 | 16 | 69.4±4.9 | 25 | MELODIC | Functional brain  networks from |
| Lottman, K.K.,  et al [29] 2017 | fMRI | SP | 34 | 32.4 ± 10.4 | 35 | 32.0 ± 8.9 | 100 | GIFT toolbox | Functional brain  networks from |
| Du, Y., et al  [30] 2017 | fMRI | Schizophrenia | 53 CHR, 58 ESZ | 20.4 ± 4.5,  21.8 ± 3.8 | 70 | 21.9 ± 5.6 | 30 | GIG-ICA | Functional brain  networks from ICA |
| Wang, L.,  et al.[31] 2018 | fMRI | Internetgamingdisorder(IGD) | 18 | 21.5 ± 2.0 | 19 | 22.3 ± 1.8 | 20 | GIFT toolbox | Functional brain  networks from ICA |
| Osuch, E., et al [32] 2018 | fMRI | Mood disorder diagnosis | BD32,  MDD34 | 21.3 ± 2.9 | 33 | 20.2 ± 2.0 | 20 | GIFT toolbox | Functional brain  networks from ICA |
| Zhang, S.,  et al.[33] 2018 | fMRI | MDD | 53 (35,18) | 20.6 ± 3.7,  21.3 ± 3.0 | 47 | 20.5 ± 1.9 | 40 | MICA toolbox | Functional brain  networks from ICA |
| Fu, Z., et al  [34] 2018 | fMRI | SP | 151 | 37.8 ± 11.4 | 163 | 36.9 ± 11.0 | 100 | GIFT toolbox | Calculate dALFF and dALFF-dFC correlations. |
| Qiao, J., et al.  [35] 2018 | fMRI | AD | 34 | 68.6 ± 9.9 | 34 | 68.6 ± 9.9 | 20-120 | GIFT toolbox | 3LHPM-ICA method, Functional brain  networks from ICA |
| Wu, L., et al.  [36] 2018 | fMRI | SP | 60. | 38.6 ± 13.4 | 61 | 35.0 ± 10.5 | 50 | GIFT toolbox | Functional brain  networks from ICA |
| Zhu, X., et al.  [37] 2018 | fMRI | alcohol use disorder (AUD) | 46 | 40.4 ± 9.7 | 46 | 32.0 ± 8.9 | 32 | MELODIC | Functional brain  networks from ICA |
| Su, J., et al  [38] 2019 | fMRI | CADASIL | 22 | 48.9 ± 14.2 | 44 | 48.4 ± 13.7 | 31 | GIFT toolbox | Functional brain  networks from ICA |
| Díez-Cirarda, M., et al.[39] 2018 | fMRI | Parkinson | 35 (12,23) | 65.2 ± 8.3,  69.2 ± 4.5 | 26 | 68.3 ± 7.5 | 100 | GIFT toolbox | Network-based statistic (NBS) approach |
| Xiao, F.,  et al.[40] 2019 | MRI | Narcolepsy | 26 | 25.8 ± 6.6 | 30 | 25.4 ± 4.3 | 48 | GIFT toolbox | Functional brain  networks from ICA |
| Fiorenzato, E.,  et al.,[41] 2019 | fMRI | Parkinson | 118 (52,46,20) | 58.6 ± 9.9,  65.9 ± 11.4,  71.8 ± 6.6 | 35 | 61.3 ± 8.9 | 120 | GIFT toolbox | Sliding window approach |
| Salman, M.S., et al [42] 2019 | fMRI | SP | 87 | / | 100 | / | 100 | GIFT toolbox | Functional brain  networks from ICA |
| Lin, H., et al.  [43] 2020 | fMRI | MDD | 59 | 57.6 ± 10.7  (41.0 – 75.0) | 97 | 61.2 ± 9.5 (44.0 - 79.0) | 100 | MELODIC | Functional brain  networks from ICA |
| Navalpotro-Gomez, I., et al.  [44] 2020 | fMRI | Parkinson | 36  (16, 20) | 61.3 ± 8.2,  63.5 ± 8.1 | 17 | 63.5 ± 9.7 | 100 | GIFT toolbox | Sliding window and graph-theory analyses |
| Jiao, K., et al.,  [45] 2020 | fMRI | MDD | 38  (19, 19) | / | 19 | / | **47, 49, and 50respectively** | GIFT toolbox | Functional brain  networks from ICA |
| Cai, X.L.,  et al.[46] 2020 | fMRI | SP | 51 | 43.2 ± 10.9 | 51 | 42.0 ± 12.2 | 30 | GIFT toolbox | Functional brain  networks from ICA |
| Gürsel, D.A., et al [47] 2020 | fMRI | Obsessive–compulsive disor-  der | 42 | 34.4 ± 12.1 | 50 | 35.1 ± 10.0 | 20 | GIFT toolbox | Sliding time window analysis |

**Reference**

[1] Greicius, M.D., G. Srivastava, A.L. Reiss, et al., Default-mode network activity distinguishes Alzheimer\"s disease from healthy aging: Evidence from functional MRI[J]*.* Proceedings of the National Academy of Sciences of the United States of America. 101(13): p. 4637-4642.

[2] Sorg, C., V. Riedl, M. Mühlau, et al., Selective changes of resting-state networks in individuals at risk for Alzheimer's disease[J]*.* Proceedings of the National Academy of Sciences of the United States of America, 2007. 104(47): p. 18760-18765.

[3] Greicius, M.D., B.H. Flores, V. Menon, et al., Resting-State Functional Connectivity in Major Depression: Abnormally Increased Contributions from Subgenual Cingulate Cortex and Thalamus[J]*.* Biol Psychiatry, 2007. 62(5): p. 429-437.

[4] Calhoun, V.D., K.A. Kiehl, and G.D. Pearlson, Modulation of Temporally Coherent Brain Networks Estimated Using ICA at Rest and During Cognitive Tasks[J]*.* Human Brain Mapping, 2008. 29(7): p. 828-838.

[5] Sui, J., T. Adali, G.D. Pearlson, et al., An ICA-based method for the identification of optimal FMRI features and components using combined group-discriminative techniques[J]*.* 46(1): p. 73-86.

[6] Filippini, N., B.J. Macintosh, M.G. Hough, et al., Distinct patterns of brain activity in young carriers of the APOE-ε4 allele[J]*.* 2009. 47(17): p. S139-S139.

[7] Temporal lobe and “default” hemodynamic brain modes discriminate between schizophrenia and bipolar disorder[J]*.* Human Brain Mapping, 2009. 29(11): p. 1265-1275.

[8] Veer, I.M., C. Beckmann, M.-J. Van Tol, et al., Whole brain resting-state analysis reveals decreased functional connectivity in major depression[J]*.* Frontiers in systems neuroscience, 2010. 4: p. 41.

[9] Fan, Y., Y. Liu, H. Wu, et al., Discriminant analysis of functional connectivity patterns on Grassmann manifold[J]*.* Neuroimage, 2011. 56(4): p. 2058-2067.

[10] Ahmed, A.E., L. Harri, R. Jukka, et al., Group-ICA Model Order Highlights Patterns of Functional Brain Connectivity[J]*.* Frontiers in Systems Neuroscience. 5.

[11] Petrella, J.R., F.C. Sheldon, S.E. Prince, et al., Default mode network connectivity in stable vs progressive mild cognitive impairment[J]*.* Neurology, 2011. 76(6): p. 511-517.

[12] Binnewijzend, M.A., M.M. Schoonheim, E. Sanz-Arigita, et al., Resting-state fMRI changes in Alzheimer's disease and mild cognitive impairment[J]*.* Neurobiology of aging, 2012. 33(9): p. 2018-2028.

[13] Du, W., V.D. Calhoun, H. Li, et al., High classification accuracy for schizophrenia with rest and task fMRI data[J]*.* Frontiers in human neuroscience, 2012. 6: p. 145.

[14] Damoiseaux, J.S., K.E. Prater, B.L. Miller, et al., Functional connectivity tracks clinical deterioration in Alzheimer[U+05F3]s disease[J]*.* Neurobiology of Aging, 2012. 33(828): p. e819-830.

[15] Fan, T., Y. Li, W. Xia, et al. Independent component analysis of the resting-state brain functional MRI study in adults with bipolar depression[C]. in *Complex Medical Engineering (CME), 2012 ICME International Conference on*. 2012.

[16] Arbabshirani, M.R., K.A. Kiehl, G.D. Pearlson, et al., Classification of schizophrenia patients based on resting-state functional network connectivity[J]*.* Frontiers in Neuroscience. 7.

[17] Vanessa, S., S. Kaustubh, B. Juan, et al., A Robust Classifier to Distinguish Noise from fMRI Independent Components[J]*.* Plos One. 9(4): p. e95493-.

[18] Hoekzema, E., S. Carmona, J.A. Ramos-Quiroga, et al., An independent components and functional connectivity analysis of resting state fMRI data points to neural network dysregulation in adult ADHD[J]*.* Hum Brain Mapp, 2014. 35(4): p. 1261-72.

[19] Maneshi, M., S. Vahdat, F. Fahoum, et al., Specific Resting-State Brain Networks in Mesial Temporal Lobe Epilepsy[J]*.* Frontiers in Neurology, 2014. 5.

[20] Kaufmann, T., K.C. Skåtun, D. Alnæs, et al., Disintegration of sensorimotor brain networks in schizophrenia[J]*.* Schizophrenia bulletin, 2015. 41(6): p. 1326-1335.

[21] Baggio, H.C., B. Segura, R. Sala-Llonch, et al., Cognitive impairment and resting-state network connectivity in Parkinson's disease[J]*.* Hum Brain Mapp, 2015. 36(1): p. 199-212.

[22] Tessitore, A., A. Giordano, R. De Micco, et al., Functional connectivity underpinnings of fatigue in "Drug-Naïve" patients with Parkinson's disease[J]*.* Mov Disord, 2016. 31(10): p. 1497-1505.

[23] He, H., Q. Yu, Y. Du, et al., Resting-state functional network connectivity in prefrontal regions differs between unmedicated patients with bipolar and major depressive disorders[J]*.* J Affect Disord, 2016. 190: p. 483-493.

[24] Yang, W., X. Chen, D.S. Cohen, et al., Classification of MRI and psychological testing data based on support vector machine[J]*.* International journal of clinical and experimental medicine, 2017. 10(12): p. 16004.

[25] Qureshi, M.N.I., J. Oh, D. Cho, et al., Multimodal discrimination of schizophrenia using hybrid weighted feature concatenation of brain functional connectivity and anatomical features with an extreme learning machine[J]*.* Frontiers in neuroinformatics, 2017. 11: p. 59.

[26] Skåtun, K.C., T. Kaufmann, N.T. Doan, et al., Consistent functional connectivity alterations in schizophrenia spectrum disorder: a multisite study[J]*.* Schizophrenia bulletin, 2017. 43(4): p. 914-924.

[27] Wu, X.J., L.-L. Zeng, H. Shen, et al., Functional network connectivity alterations in schizophrenia and depression[J]*.* Psychiatry Research Neuroimaging. 263: p. 113-120.

[28] Komal, B., B. Matteo, U. Neeraj, et al., Abnormal Resting-State Functional Connectivity in Progressive Supranuclear Palsy and Corticobasal Syndrome[J]*.* Front Neurol, 2017. 8: p. 248-.

[29] Lottman, K.K., N.V. Kraguljac, D.M. White, et al., Risperidone Effects on Brain Dynamic Connectivity-A Prospective Resting-State fMRI Study in Schizophrenia[J]*.* Front Psychiatry, 2017. 8: p. 14.

[30] Du, Y., S.L. Fryer, D. Lin, et al., Identifying functional network changing patterns in individuals at clinical high-risk for psychosis and patients with early illness schizophrenia: A group ICA study[J]*.* Neuroimage Clinical, 2017. 17(C): p. 335-346.

[31] Wang, L., Y. Zhang, X. Lin, et al., Group independent component analysis reveals alternation of right executive control network in Internet gaming disorder[J]*.* CNS spectrums, 2018. 23(5): p. 300-310.

[32] Osuch, E., S. Gao, M. Wammes, et al., Complexity in mood disorder diagnosis: fMRI connectivity networks predicted medication-class of response in complex patients[J]*.* Acta Psychiatr Scand, 2018. 138(5): p. 472-482.

[33] Zhang, S., J.-m. Chen, L. Kuang, et al., Association between abnormal default mode network activity and suicidality in depressed adolescents[J]*.* Bmc Psychiatry, 2018. 16(1): p. 337.

[34] Fu, Z., Y. Tu, X. Di, et al., Characterizing dynamic amplitude of low-frequency fluctuation and its relationship with dynamic functional connectivity: an application to schizophrenia[J]*.* Neuroimage, 2018. 180: p. 619-631.

[35] Qiao, J., Y. Lv, C. Cao, et al., Multivariate Deep Learning Classification of Alzheimer’s Disease Based on Hierarchical Partner Matching Independent Component Analysis[J]*.* Frontiers in aging neuroscience, 2018. 10: p. 417.

[36] Wu, L., A. Caprihan, J. Bustillo, et al., An approach to directly link ICA and seed-based functional connectivity: Application to schizophrenia[J]*.* Neuroimage, 2018. 179: p. 448-470.

[37] Zhu, X., X. Du, M. Kerich, et al., Random forest based classification of alcohol dependence patients and healthy controls using resting state MRI[J]*.* Neuroscience letters, 2018. 676: p. 27-33.

[38] Su, J., S. Ban, M. Wang, et al., Reduced resting-state brain functional network connectivity and poor regional homogeneity in patients with CADASIL[J]*.* J Headache Pain, 2019. 20(1): p. 103.

[39] Díez-Cirarda, M., A.P. Strafella, J. Kim, et al., Dynamic functional connectivity in Parkinson's disease patients with mild cognitive impairment and normal cognition[J]*.* Neuroimage Clin, 2018. 17: p. 847-855.

[40] Xiao, F., C. Lu, D. Zhao, et al., Independent component analysis and graph theoretical analysis in patients with narcolepsy[J]*.* Neuroscience bulletin, 2019. 35(4): p. 743-755.

[41] Fiorenzato, E., A.P. Strafella, J. Kim, et al., Dynamic functional connectivity changes associated with dementia in Parkinson's disease[J]*.* Brain, 2019. 142(9): p. 2860-2872.

[42] Salman, M.S., Y. Du, D. Lin, et al., Group ICA for identifying biomarkers in schizophrenia: 'Adaptive' networks via spatially constrained ICA show more sensitivity to group differences than spatio-temporal regression[J]*.* Neuroimage Clin, 2019. 22: p. 101747.

[43] Lin, H., X. Cai, D. Zhang, et al., Functional connectivity markers of depression in advanced Parkinson's disease[J]*.* NeuroImage: Clinical, 2020. 25: p. 102130.

[44] Navalpotro-Gomez, I., J. Kim, P.M. Paz-Alonso, et al., Disrupted salience network dynamics in Parkinson's disease patients with impulse control disorders[J]*.* Parkinsonism Relat Disord, 2020. 70: p. 74-81.

[45] Jiao, K., H. Xu, C. Teng, et al., Connectivity patterns of cognitive control network in first episode medication-naive depression and remitted depression[J]*.* Behav Brain Res, 2020. 379: p. 112381.

[46] Cai, X.L., D.J. Xie, K.H. Madsen, et al., Generalizability of machine learning for classification of schizophrenia based on resting‐state functional MRI data[J]*.* Human Brain Mapping, 2020. 41(1): p. 172-184.

[47] Gürsel, D.A., L. Reinholz, B. Bremer, et al., Frontoparietal and salience network alterations in obsessive–compulsive disorder: insights from independent component and sliding time window analyses[J]*.* J Psychiatry Neurosci, 2020. 45(3): p. 190038.
