# Supplementary material for: Construction and Multiple Feature Classification Based on a High-Order Functional Hypernetwork on fMRI Data
Source: Front Neurosci. 2022 Apr 13;16:848363. doi: 10.3389/fnins.2022.848363 (PMC9043754; doi:10.3389/fnins.2022.848363)
Supplement: Supplementary file 7 [file Table_2.DOCX]

**Supplemental Table S2. Peak activations of ICN spatial maps.**

| **ICNs** |  |  | Coordinate | | |  |  | **ICNs** |  |  | Coordinate | | |
| --- | --- | --- | --- | --- | --- | --- | --- | --- | --- | --- | --- | --- | --- |
|  |  | $t_{\max}$ | X | Y | Z |  |  |  |  | $t_{\max}$ | X | Y | Z |
| **Auditory networks** |  |  |  |  |  |  |  | IC 31(0.97) |  |  |  |  |  |
| IC 15(0.97) |  |  |  |  |  |  |  | Temporal_Mid_L | 177 | 31.5 | -63 | -45 | -6 |
| Temporal_Sup_L | 566 | 53.1 | -63 | -18 | 9 |  |  | ParaHippocampal_L | 74 | 29 | -15 | -9 | -27 |
| Temporal_Sup_R | 530 | 53.1 | 60 | -21 | 12 |  |  | Lingual_L | 38 | 20.4 | -21 | -93 | -18 |
| **Sensorimotor networks** |  |  |  |  |  |  |  | **Attentional networks** |  |  |  |  |  |
| IC 4(0.99) |  |  |  |  |  |  |  | IC 24(0.98) |  |  |  |  |  |
| Postcentral_R | 545 | 39.6 | 45 | -33 | 63 |  |  | Precuneus_L | 425 | 41.4 | -3 | -66 | 60 |
| Postcentral_L | 61 | 22.7 | -30 | -27 | 75 |  |  | Frontal_Mid_L | 208 | 35.8 | -30 | 36 | 48 |
| IC 8(0.98) |  |  |  |  |  |  |  | Temporal_Mid_R | 29 | 12.8 | 66 | -33 | 12 |
| Precentral_L | 311 | 32.9 | -27 | -21 | 75 |  |  | IC 25(0.97) |  |  |  |  |  |
| Supp_Motor_Area_L | 23 | 20 | -3 | -9 | 54 |  |  | Frontal_Mid_Orb_L | 119 | 32.8 | -45 | 45 | -9 |
| IC 11(0.98) |  |  |  |  |  |  |  | SupraMarginal_R | 83 | 25.4 | 63 | -27 | 21 |
| Paracentral_Lobule_L | 313 | 35.5 | -8 | -25 | 70 |  |  | Temporal_Pole_Sup_L | 53 | 20.1 | -51 | 18 | -9 |
| Paracentral_Lobule_R | 206 | 33 | 7 | -32 | 68 |  |  | Lingual_L | 40 | 18.1 | -12 | -93 | -15 |
| Insula_L | 49 | 20.6 | -33 | -24 | 15 |  |  | IC 30(0.96) |  |  |  |  |  |
| IC 22(0.98) |  |  |  |  |  |  |  | Angular_R | 420 | 48.1 | 45 | -60 | 54 |
| SupraMarginal_R | 423 | 44.4 | 63 | -33 | 36 |  |  | Parietal_Inf_L | 202 | 35.7 | -45 | -54 | 57 |
| SupraMarginal_L | 202 | 29.5 | -63 | -33 | 30 |  |  | Frontal_Mid_R | 180 | 29.3 | 36 | 15 | 60 |
| Frontal_Mid_L | 105 | 29.4 | -45 | 42 | 21 |  |  | Cingulum_Mid_R | 82 | 25.1 | 3 | -36 | 45 |
| Cingulum_Mid_L | 55 | 21.4 | 0 | 12 | 36 |  |  | Frontal_Sup_Medial_R | 63 | 22.1 | 3 | 33 | 42 |
| Paracentral_Lobule_R | 21 | 15.1 | 3 | -36 | 51 |  |  | IC 35(0.96) |  |  |  |  |  |
| IC 36(0.96) |  |  |  |  |  |  |  | Angular_R | 316 | 39.7 | 42 | -75 | 39 |
| Supp_Motor_Area_R | 81 | 28.7 | 9 | 0 | 62 |  |  | Temporal_Mid_L | 181 | 29.9 | -60 | -9 | -21 |
| Supp_Motor_Area_L | 63 | 26.6 | -5 | 5 | 61 |  |  | Precuneus_R | 117 | 25.8 | 15 | -54 | 15 |
| Precentral_R | 21 | 15.2 | 21 | -18 | 78 |  |  | Frontal_Med_Orb_L | 28 | 11.8 | 0 | 51 | -9 |
| **Visual networks** |  |  |  |  |  |  |  | IC 39(0.96) |  |  |  |  |  |
| IC 10(0.98) |  |  |  |  |  |  |  | Angular_L | 286 | 40 | -45 | -66 | 48 |
| Occipital_Inf_R | 231 | 33 | 30 | -96 | -3 |  |  | Frontal_Mid_L | 190 | 30.1 | -30 | 24 | 57 |
| Occipital_Inf_L | 215 | 33 | -27 | -96 | -6 |  |  | Angular_R | 137 | 27.1 | 51 | -60 | 42 |
| IC 19(0.98) |  |  |  |  |  |  |  | Cingulum_Mid_L | 48 | 18.8 | -3 | -39 | 36 |
| Calcarine_R | 432 | 33.6 | 18 | -57 | 6 |  |  | Precuneus_R | 35 | 17.9 | 6 | -66 | 60 |
| Occipital_Mid_R | 19 | 22.8 | 30 | -93 | 12 |  |  | IC 40(0.93) |  |  |  |  |  |
| IC 32(0.97) |  |  |  |  |  |  |  | Parietal_Sup_R | 118 | 28.3 | -23 | -60 | 59 |
| Lingual_R | 109 | 30.4 | -15 | -68 | -5 |  |  | Frontal_Sup_Medial_L | 40 | 26 | -6 | 66 | 18 |
| Lingual_L | 94 | 30.4 | 16 | -67 | -4 |  |  | **Frontal networks** |  |  |  |  |  |
| IC 34(0.97) |  |  |  |  |  |  |  | IC 33(0.97) |  |  |  |  |  |
| Temporal_Mid_R | 439 | 38.9 | 54 | -69 | 3 |  |  | Frontal_Inf_Tri_L | 463 | 47.7 | -51 | 21 | 30 |
| Occipital_Mid_L | 153 | 20.5 | -51 | -75 | 6 |  |  | Parietal_Inf_L | 191 | 38.2 | -33 | -72 | 48 |
| IC 38(0.93) |  |  |  |  |  |  |  | Angular_R | 122 | 37.7 | 57 | -57 | 36 |
| Cuneus_L | 247 | 34.8 | -6 | -80 | 27 |  |  | Temporal_Inf_L | 60 | 26.8 | -60 | -54 | -9 |
| Cuneus_R | 226 | 20.2 | 14 | -79 | 28 |  |  | Frontal_Sup_Medial_L | 36 | 24.3 | -3 | 30 | 48 |
| **Default mode networks** |  |  |  |  |  |  |  | Parietal_Sup_R | 32 | 23.9 | 18 | -66 | 66 |
| IC 16(0.98) |  |  |  |  |  |  |  | IC 43(0.93) |  |  |  |  |  |
| Precuneus_L | 532 | 35.4 | 0 | -75 | 39 |  |  | Frontal_Inf_Orb_L | 121 | 34.3 | -48 | 21 | -9 |
| Angular_R | 39 | 20.5 | 39 | -60 | 48 |  |  | Temporal_Mid_R | 116 | 37.7 | 51 | -33 | -3 |
| IC 18(0.98) |  |  |  |  |  |  |  | Frontal_Sup_Medial_L | 91 | 31.7 | -3 | 57 | 24 |
| Lingual_L | 44 | 27.6 | -24 | -93 | -18 |  |  | Angular_R | 75 | 27.1 | 63 | -51 | 27 |
| Frontal_Sup_Medial_L | 42 | 24.2 | 0 | 60 | 3 |  |  | Precuneus_L | 68 | 26.8 | -3 | -54 | 42 |

The quality index ($I_{q}$) associated with each ICN is listed in parentheses adjacent to the component number. , number of voxels in each cluster; $t_{\max}$, maximum t-statistic in each component; Coordinate, coordinate (in mm) of $t_{\max}$ in MNI space, following LPI convention.
