# Supplementary material for: Construction and Multiple Feature Classification Based on a High-Order Functional Hypernetwork on fMRI Data
Source: Front Neurosci. 2022 Apr 13;16:848363. doi: 10.3389/fnins.2022.848363 (PMC9043754; doi:10.3389/fnins.2022.848363)
Supplement: Supplementary file 8 [file Table_3.DOCX]

**Supplemental Table S3. Classification results of ablation experiments.**

| **Removing Local property** | **Accuracy (%)** | **Sensitivity (%)** | **Specificity (%)** | **BAC (%)** |
| --- | --- | --- | --- | --- |
| **HCC^1^** | 87.54 | 94.74 | 77.78 | 86.26 |
| **HCC^2^** | 86.95 | 93.13 | 78.57 | 85.85 |
| **HCC**^3^ | 80.89 | 89.47 | 69.25 | 79.36 |
| **SP** | 82.49 | 91.96 | 69.64 | 80.80 |
| **HCCPN** | 88.64 | 93.71 | 81.75 | 87.73 |

HCC1 represents the first type of hypernetwork clustering coefficient based on a single node; HCC2 represents the second type of hypernetwork clustering coefficient based on a single node; HCC3 represents the third type of hypernetwork clustering coefficient based on a single node; SP represents the shortest path. HCCPN represents the hypernetwork clustering coefficient based on pairs of nodes.
