# Supplementary material for: Construction and Multiple Feature Classification Based on a High-Order Functional Hypernetwork on fMRI Data
Source: Front Neurosci. 2022 Apr 13;16:848363. doi: 10.3389/fnins.2022.848363 (PMC9043754; doi:10.3389/fnins.2022.848363)
Supplement: Supplementary file 9 [file Table_4.docx]

**Supplemental Table S4. The classification accuracy of different methods relating to the AD dataset.**

| **Method** | **Research** | **Accuracy (%)** | **Sensitivity (%)** | **Specificity (%)** | **BAC (%)** |
| --- | --- | --- | --- | --- | --- |
| **TBFN** | Fusion feature | 79.66 | 79.31 | 80.00 | 79.66 |
| **rs-HOFN** | Fusion feature | 90.91 | 92.11 | 89.29 | 90.70 |
| **rs-FHN** | Fusion feature | 90.15 | 94.74 | 85.71 | 90.23 |
| **rs-HOFHN** | Fusion feature | 93.18 | 94.74 | 92.86 | 93.80 |

TBFN represents traditional simple binary functional network model; rs-HOFN represents resting state high-order functional network; rs-FHN represents resting state functional hypernetwork; rs-HOFHN represents resting state high-order functional hypernetwork. BAC represents balanced accuracy. Fusion feature represents local properties combined with subgraph feature. AD represents patients with Alzheimer’s disease
